# Spherical radiomics for radiogenomic assessment of glioblastoma heterogeneity

**DOI:** 10.1093/noajnl/vdag132

**Published:** 2026-05-16

**Authors:** Haotian Feng, Ke Sheng

**Affiliations:** Department of Radiation Oncology, University of California, San Francisco, CA, USA; Department of Radiation Oncology, University of California, San Francisco, CA, USA

**Keywords:** glioblastoma, machine learning, radiogenomic, spherical radiomics, tumor heterogeneity

## Abstract

**Background:**

To develop and validate a novel spherical radiomics framework for predicting key molecular biomarkers—including MGMT promoter methylation, EGFR, and PTEN mutation status—and survival in glioblastoma (GBM) patients using multiparametric MRI.

**Methods:**

Using the UCSF-PDGM cohort, we propose a spherical radiomics framework in which tumor-centered concentric shells are generated at increasing radial distances from the tumor centroid and mapped onto two-dimensional surfaces for feature extraction. Radiomic features—including shape, first-order statistics, and texture descriptors (GLCM, GLRLM, GLDM, GLSZM, and NGTDM)—were extracted from 299 GBM patients using PyRadiomics across 4 tumor regions: necrotic core, T1-weighted contrast-enhancing region, T2/FLAIR hyperintense lesion, and a 2 cm peritumoral expansion region. Classification was performed using multiple machine learning models, including neural networks, logistic regression, random forest, and the Tree-based Pipeline Optimization Tool (TPOT). The proposed framework was further validated on an external UPENN-GBM cohort. Model interpretability was assessed using SHAP analysis, feature significance profiling, clustering visualization, and evaluation of radiomic patterns in relation to underlying biological processes. Radial transition analysis was conducted to quantify feature changes across adjacent tumor regions.

**Results:**

Spherical radiomics achieved AUCs of 0.82 for MGMT, 0.77 for EGFR, 0.76 for PTEN, and 0.79 for survival prediction, consistently outperforming conventional Euclidean radiomics (0.71, 0.61, 0.70, and 0.61, respectively). Consistent improvements were also observed in external validation based on UPENN-GBM cohort, with an AUC improved by 8% compared to Euclidean radiomics. GLCM-derived features were identified as the most informative predictors. Radial transition analysis using the Mann-Whitney U-test demonstrated that transition slopes between the T1-weighted contrast-enhancing and T2/FLAIR hyperintense regions, as well as between the T2/FLAIR hyperintense and peritumoral regions, were significantly associated with biomarker status.

**Conclusion:**

Radiomic features extracted from spherical surfaces at varying radial distances from the tumor centroid demonstrate stronger associations with key molecular markers and patient survival compared to conventional Euclidean radiomics, highlighting the value of spatially structured radiomic analysis for improved GBM characterization.

Key Points

- Radiomics derived in Euclidean space fails to capture the structural complexity of GBM.
- Spherical Radiomics more accurately reflects the evolutionary patterns of GBM and demonstrates substantially improved prediction of biomarkers and patient survival.

Importance of the Study

We develop and rigorously validate a novel spherical radiomics framework for the quantitative characterization of GBM using multiparametric MRI. In contrast to conventional Euclidean radiomics, which extract features on fixed orthogonal grids, our method computes features on concentric spherical shells centered at the tumor centroid, thereby capturing biologically meaningful radial growth and infiltration patterns that better reflect tumor architecture. Based on 299 patients’ multiparametric MRI, we demonstrate that the proposed framework substantially enhances the prediction accuracy of critical molecular biomarkers—including MGMT promoter methylation, EGFR, and PTEN mutations—as well as patient survival, when compared with traditional 2D and 3D Euclidean analyses. Moreover, our findings reveal that radial texture transitions within the tumor strongly correlate with its molecular profile, suggesting that the spatial organization of radiomic heterogeneity encodes underlying biological processes driving GBM evolution and progression. This work establishes spherical radiomics as a powerful and generalizable paradigm for radiogenomic analysis.

Glioblastoma (GBM) is the most aggressive and lethal primary brain tumor in adults, accounting for approximately 16% of all primary brain and central nervous system neoplasms, with an age-adjusted incidence rate of 3.2 per 100 000 population.^1^ Although GBMs occur almost exclusively in the brain, they can also arise in the brainstem, cerebellum, and spinal cord.^2^ Among primary gliomas, 61% occur in the cerebral lobes, most commonly in the frontal (25%), temporal (20%), parietal (13%), and occipital (3%) regions.^3^ GBM arises from glial cells—the supportive cells of the central nervous system—and is characterized by rapid proliferation, diffuse infiltration, and resistance to standard therapies. Despite advances in surgical resection, radiotherapy, and chemotherapy, median survival remains only 14-18 months.^4^

One major contributor to this poor prognosis is the profound molecular and phenotypic heterogeneity of GBM at the genetic, cellular, and radiographic levels.^5^ Intertumoral heterogeneity refers to differences in molecular, genetic, or imaging features between tumors from different patients, whereas intratumoral heterogeneity describes diversity within a single tumor mass. GBM frequently exhibits marked intratumoral heterogeneity, which influences treatment response, tumor progression, and overall outcomes.^6^ This heterogeneity is evident in the spatial distribution of proliferative, hypoxic, and necrotic regions, as well as in the molecular landscape, where biomarker expressions such as MGMT and EGFR can vary across different tumor subregions.^7^ Such diversity has direct clinical implications, including precise diagnosis, identification of actionable driver mutations, and clinical trial design.^8^ Intratumoral heterogeneity can be categorized at the molecular,^9^^,^^10^ cellular,^11^ and tissue^12^ levels, but also poses significant challenges for tissue sampling and biopsy-based molecular testing.^13^^,^^14^

Given the limitations of invasive brain biopsies in capturing tumor heterogeneity, radiogenomics has emerged as a powerful noninvasive method to correlate quantitative imaging features with underlying genomic profiles.^15^ By capturing whole tumor phenotypes that reflect biological processes such as cellularity, angiogenesis, and necrosis,^16^ radiogenomics can provide complementary insights to molecular testing. Furthermore, radiomics features extracted from 2D or 3D multimodal imaging have been used to predict MGMT promoter methylation,^17^^,^^18^ EGFRvIII mutation,^19^^,^^20^ PTEN,^21^ and survival status^22^ with varying accuracies.

Traditional radiomics approaches—whether based on 2D slices or entire 3D tumor volumes—are based on Euclidean geometry, which are not intrinsic to the tumor growth pattern. Mathur et al^23^ recently showed that GBM intratumoral heterogeneity demonstrates a strong and statistically significant dependence on the distance to the tumor centroid. For example, the classical and mesenchymal cells are more centrally located vs. the more peripherally located proneuronal and neural subtypes. Also, as shown by Greenwald et al,^24^ GBM comprises both disorganized and structured regions, with the structured regions exhibiting a five-layered spatial organization primarily driven by hypoxia. These geometrical patterns are consistent with tumor radial growth and evolution. However, existing radiomics analysis based on Euclidean geometry is insensitive to the radially evolving patterns, which requires a new approach based on spherical coordinates.

In this study, we propose spherical radiomics that extracts features from concentric shells of increasing radii from the tumor centroid to enhance the structural representation of image heterogeneity that is consistent with tumor evolution.

## Overview of Research Approach

**Figure 1.**
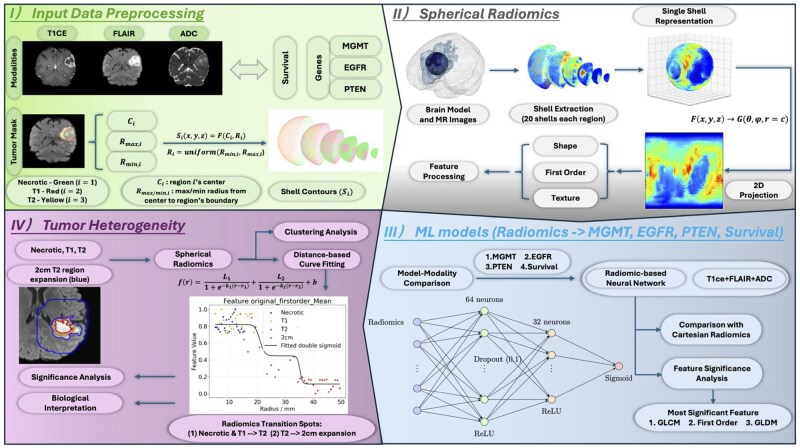
Study flowchart: (I) Input data consisted of 3 imaging modalities (T1CE, FLAIR, and ADC) along with molecular markers (MGMT promoter methylation, EGFR and PTEN mutation status) and survival status. Tumor masks were divided into 3 subregions: necrotic core, enhancing tumor (T1), and edema/infiltrative region (T2). Shell masks were generated using the tumor centroid and the minimum and maximum radii encompassing each subregion. (II) Imaging intensities were interpolated onto the shell masks and projected onto 2D planes. Radiomic features, including shape features from the 3D tumor region, first-order statistics, and multiple texture categories from projected 2D planes, were then extracted from each plane. An ANOVA F-test was applied to select the most discriminative features. (III) Multiple machine learning models and modality combinations were tested. The neural network framework and logistic regression model integrating all modalities achieved the highest predictive performance. Feature importance analysis further revealed that GLCM-based features were the most predictive for different molecular and survival statuses. (IV) To characterize the radiomic gradient beyond the visible tumor boundary, we generated a 2 cm isotropic expansion of the T2 region. Spherical radiomic features extracted from this peritumoral region, together with those from the original tumor regions, were modeled as a function of radius (distance from the tumor center). The transition slope from the tumor core to the surrounding tissue emerged as a significant parameter, reflecting underlying biological processes. Furthermore, clustering analysis highlighted the advantage of spherical radiomics in capturing and explaining tumor heterogeneity.

An overview of this research work is summarized and described in Figure 1. The tumor masks for medical images are generated using an ensemble model consisting of prior BraTS challenge-winning segmentation algorithms and then manually corrected by trained radiologists and approved by 2 expert reviewers.^25^ Then, spherical radiomics are extracted from each tumor region and further used to develop machine learning (ML) models for predicting molecular and survival status.

In this study, we primarily utilized the dataset UCSF-PDGM, which is a publicly available cohort that includes imaging and molecular data from 501 GBM patients in TCIA (299 valid GBM patients with required molecular and survival information).^25^ A demographic description of the patient cohorts is provided in Supplementary A, Table S1. For UCSF-PDGM datasets, we focused on T1-weighted contrast-enhanced (T1CE), FLAIR, and Apparent Diffusion Coefficient (ADC) MRI sequences. T1CE and FLAIR are routinely acquired in standard neuro-oncology workflows and provide complementary information on tumor enhancement and peritumoral edema. ADC, derived from diffusion-weighted imaging, reflects water molecule diffusivity and serves as a surrogate marker for tumor cellularity, offering valuable physiological information that can enhance radiogenomic characterization.

An external validation of the model was conducted utilizing the UPENN-GBM dataset^26^ with an independent cohort of 87 GBM patients with confirmed MGMT status. Only T1CE and FLAIR were utilized for external validation as ADC data were not available in UPENN-GBM. The demographic description of UPENN-GBM is provided in Supplementary A, Table S2.

## Methods

### Different Tumor Regions of GBM

GBM typically exhibits four anatomically and biologically distinct regions identifiable on MRI: the necrotic core, the T1-weighted contrast-enhancing region, the T2/FLAIR hyperintense lesion, and the 2cm expansion region.^27^ The necrotic core, usually visualized as a non-enhancing central area, reflects hypoxic tissue death and is associated with aggressive tumor biology and poor prognosis. Surrounding this core is the contrast-enhancing rim on post-contrast T1-weighted images, which indicates regions of high vascular permeability and blood-brain barrier breakdown. This area contains the bulk of proliferative tumor cells and is typically the primary target for surgical resection and radiotherapy. Beyond the enhancing rim lies the T2/FLAIR hyperintense region, which encompasses both infiltrative tumor cells and vasogenic edema. Although this region appears less aggressive radiologically, it is known to harbor microscopic tumor invasion and is frequently implicated in recurrence following treatment.

In addition to these radiographically visible regions, clinical guidelines recommend a 2 cm isotropic expansion beyond the visible tumor boundary to define the peritumoral region at risk for microscopic infiltration.^28^ This margin accounts for the highly infiltrative nature of GBM, as tumor cells can extend well beyond the enhancing core. The margin is included in the radiotherapy target volume as part of the standard GBM management protocol.^29^ In this study, the 2 cm expansion mask was generated by applying binary morphological dilation to the lesion mask, using a 3D spherical structuring element scaled according to the voxel dimensions. The 2 cm expansion region radiomics has been reported to deliver predictive values for brain tumor identification,^30^ tumor infiltration, and recurrence.^31^^,^^32^

Each of these tumor regions provides complementary biological information. By analyzing them separately through radiomic features, we could achieve a more nuanced understanding of intratumoral heterogeneity and explore its association with key molecular alterations such as O^6^-methylguanine-DNA methyltransferase (MGMT) promoter methylation, Epidermal growth factor receptor (EGFR) mutation, phosphatase and tensin homolog (PTEN) mutation, as well as survival status.

### Spherical Radiomics and Feature Extraction

In this study, we introduced a novel radiomic framework termed *spherical radiomics*, specifically designed to capture the radial growth pattern of GBM. We segmented the tumor and surrounding volumes into a series of concentric 3D shells—thin, non-overlapping layers that evolve outward from the geometric center of the tumor toward its margin. Radiomic features were then extracted independently from each shell, enabling a localized, layer-wise characterization of tumor heterogeneity. This spherical decomposition allowed us to analyze spatial gradients in texture, intensity, and shape features that may correspond to various molecular statuses and survival statuses.

#### Shell contour generation

**Figure 2.**
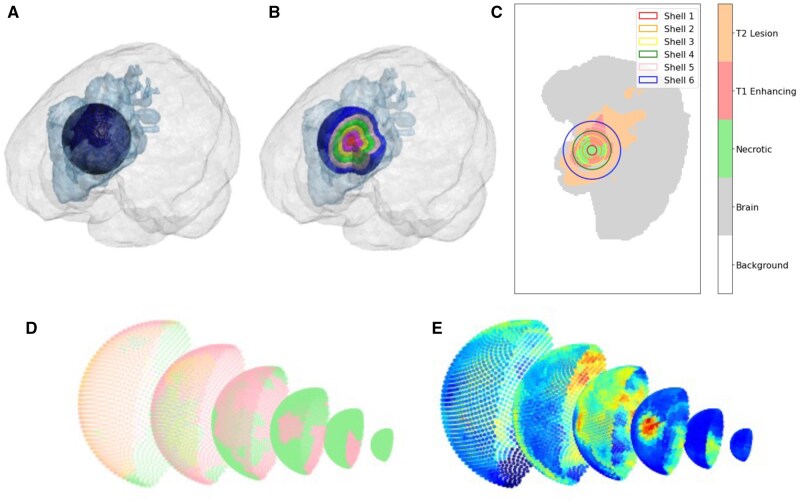
Example of spherical shell extraction in the tumor region with six shells: (A) 3D full view showing the spatial relationship between brain (light gray), tumor (light blue), and generated shells. (B) Cutout view of the spherical shells. (C) 2D slice through the tumor center. (D, E) Half spherical shell visualization with 2000 sampling points: (D) shell mask originated from tumor center, where green represents necrotic region, light red represents T1 region and orange represents T2 region (E) T1CE intensities on the corresponding shell mask.

First, shells were generated as spherical shapes centered at the center of the tumor region. Specifically, for each tumor subregion, we generated *N* uniformly spaced shells. That is, assuming *r*_max_ represented the maximum radius of the sphere shell that covers the tumor region’s outer boundary and *r*_min_ represented the minimum radius of the spherical shell that covers the tumor region’s inner boundary, the radius of the i-th spherical shell was determined as $r_{i}=r_{\min}+i\frac{r_{\max}-r_{\min}}{N}$. In this study, we chose *N* = 20, and 8000 points were sampled from each shell, and the image intensity at the sampled shell point was estimated with the nearest voxel grid in original image. It is worth noting that the sampled points could be within or outside the tumor region. An example of the extracted shell from the tumor center, as well as contour sampling and projection on shell surfaces, is shown in Figure 2. In this study, we utilized a fixed number of shells to represent the radial evolution of the tumor. We chose this approach to ensure a normalized radial representation of the tumor, consistent with the reported GBM cell pattern dependence on the normalized radial distance to the tumor center.^23^^,^^24^

#### Shell contour mapping onto 2D spherical coordinates

To extract the corresponding radiomic features using the standard radiomics formula, we mapped the shell contour onto a 2D Euclidean plane. A spherical shape is described using spherical coordinates r (radius), ϕ (azimuthal angle or longitude, in the range [−*π*, *π*]), and θ (spherical angle or colatitude, in the range [0*, π*]).

On each shell surface, we queried the corresponding pixel value of each sampled point with Equation 2. Compared to traditional 2D planes across the tumor that are insensitive to the radial transition, the projected spherical surfaces capture the radial evolution of imaging characteristics from the necrotic core to the peritumoral region. Example of mapping results, including all 20 shell layers at individual region (necrotic, T1-enhancing and T2 lesion region) is presented in Supplementary C. We adopted a 2D surface-based spherical representation, as the thickness of each shell is approximately 1 voxel (∼1 mm), making volumetric (3D) feature extraction within shells less informative and potentially unstable.


(1) [xyz]=r·[ sin φ· cos θ sin φ· sin θ cos φ]

#### Feature collection and selection

In this study, we extracted radiomic features from individual MRI modalities (T1CE, FLAIR, and ADC) as well as from their combinations to explore the benefits of multimodal integration using PyRadiomics^33^ (version 3.0.1) with image biomarker standardization initiative (IBSI)-aligned feature definitions and default settings unless otherwise specified.^34^ Prior to feature computation, all images were double checked and resampled to ensure isotropic voxel spacing of 1 × 1 × 1 mm to reduce variability arising from heterogeneous acquisition parameters. To compute texture features, voxel intensities within each region were discretized with default setting of fixed bin width of 25 intensity units, as recommended for radiomics reproducibility. Discretization was applied before the calculation of texture matrices to reduce sensitivity to noise and imaging variability. Following discretization, multiple classes of radiomic features were computed, including shape descriptors (3D tumor mask only), first-order intensity statistics, gray-label co-occurrence matrix (GLCM), gray-label dependence matrix (GLDM), gray-level run length matrix (GLRLM), gray-level size zone matrix (GLSZM), and neighboring gray-tone difference matrix (NGTDM) features.

For each image modality, a total number of 321 radiomic are extracted for 3D radiomics model, 2046 radiomic are extracted for 2D radiomic model and 16 779 radiomics are extracted for spherical radiomic model. To identify the most informative features for predicting genetic mutations and avoid overfitting, we first applied the correlation filtering and remove the highly correlated features (Spearman’s coefficient > 0.9). Later, a univariate F-test is applied on the reduced feature set to rank features based on statistical relevance to the target variable on the training set within K-fold. Features with higher F-scores indicate stronger discriminatory power and are more likely to be predictive. This approach provided an efficient means of reducing feature dimensionality and mitigating overfitting by filtering out irrelevant or noisy features. To prevent data leakage, all feature preprocessing steps, including correlation filtering and univariate feature selection, were performed exclusively on the training data within each cross-validation fold. The selected feature subsets were subsequently applied to the validation and test sets. The full CLEAR checklist^35^ is shown in Supplementary I.

#### Tumor heterogeneity description

In addition to analyzing radiomic features on individual shells, we examined tumor radiographic heterogeneity as a function of distance. For each radiomic feature, the observed profile was modeled using a double sigmoid function. This formulation integrates 2 sigmoid components, enabling representation of non-monotonic patterns such as sequential increases and decreases, or two-stage transitions, which are consistent with known spatial heterogeneity in GBM.^36^

This function, defined in Equation 2, integrates 2 sigmoid components to capture both increasing and decreasing trends, or two-stage transitions, within the feature distribution. {*L_i_*} represented the amplitudes of sigmoid function, {*x_i_*} indicated the transition point, {*k_i_*} described the slope of the transition and *b* was the constant offset term.


(2) $$f(x)=\frac{L_{1}}{1+e^{-k_{1}(x-x_{1})}}+\frac{L_{2}}{1+e^{-k_{2}(x-x_{2})}}+b$$

#### Prediction model

In this study, we implemented a fully connected three-layer neural network (NN) to predict MGMT promoter methylation, EGFR mutation status, PTEN mutation status, and patient survival status from extracted radiomic features. We utilized a median survival of 15-month progression-free survival as the efficacy endpoint of therapy trials.^37^ The network architecture comprised two hidden layers with 64 and 32 neurons, respectively, each activated with the ReLU function, followed by a sigmoid-activated output layer for binary classification. To reduce the risk of overfitting, a dropout layer with a rate of 0.1 was applied after the first hidden layer, randomly deactivating a fraction of neurons during training and thereby promoting better generalization. Model training employed the binary cross-entropy loss function. Both Adaptive Moment Estimation (Adam) and stochastic gradient descent (SGD) optimizers yielded comparable performance; in this work, we adopted SGD with a learning rate of 0.1 and a decay parameter of 0.001.

To ensure robust evaluation, the dataset was first split into training (80%) and independent test (20%) sets. A 5-fold cross-validation strategy was then performed exclusively on the training set for model development and hyperparameter tuning. The final model was evaluated on the held-out test set, and performance was reported using the area under the receiver operating characteristic curve (AUC).

The predictive performance of the NN was benchmarked against several baseline methods, including logistic regression (LR) with ridge regularization, random forest with 100 estimators, and the Tree-based Pipeline Optimization Tool (TPOT),^38^ configured with a generation size of 10 and a population size of 20. TPOT, an AutoML framework based on genetic programming, automatically optimizes ML pipelines by selecting and combining models and preprocessing steps. Across evaluations, the proposed NN had comparable performance with LR and demonstrated superior accuracy and stability compared to random forest and TPOT.

## Results

### Prediction Performance

As outlined in the research overview, we evaluated both mono- and multimodal MR radiomics and subsequently applied predictive modeling to assess the performance of the NN relative to LR, random forest (RF), and TPOT. For this study, the top 200 features were selected based on overall predictive performance for gene biomarker and survival status prediction, as shown in Supplementary D.

**Figure 3.**
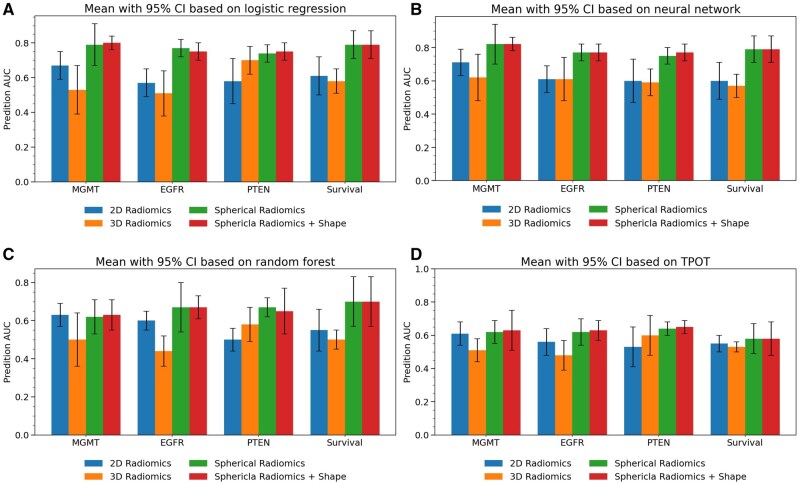
Prediction AUCs for different feature extraction methods and machine learning algorithms using all modalities: (A) Prediction based on logistic regression; (B) Prediction based on neural network; (C) Prediction based on random forest; (D) Prediction based on TPOT.

Figure 3 summarizes the predictive performance of 4 radiomic feature sets—2D radiomics, 3D radiomics, spherical radiomics, and spherical radiomics augmented with tumor mask shape features—across all 4 modeling algorithms, with the corresponding ROC curves at one-fold shown in Supplementary B. Besides, we evaluated whether adding global tumor radiomics (shape, first-order, and texture features) improved prediction performance. The analysis showed that including global features did not substantially improve model performance, suggesting that subregion-based features capture most of the predictive information.

For all prediction tasks, spherical radiomics consistently outperformed conventional 2D and 3D Euclidean feature sets. Across all modalities and model types, NN and LR consistently outperformed random forest (RF) and TPOT. For example, in predicting MGMT promoter methylation with all 3 modalities, the NN achieved the highest mean AUC of 0.82 [95% CI: 0.78-0.86], followed by LR (0.80), RF (0.63), and TPOT (0.63). DeLong testing confirmed that NN significantly outperformed RF (*P* = .01) and a trend toward improved performance compared with TPOT (*P* = .05); however, its performance was not significantly different from LR (*P* = .46).

**Figure 4.**
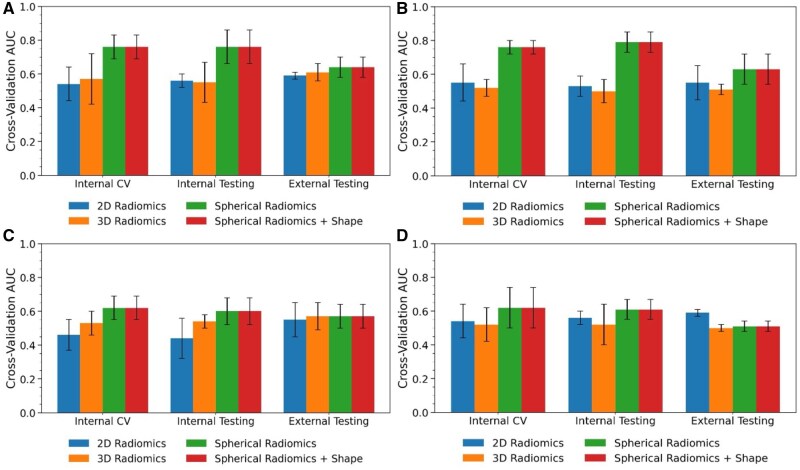
External validation prediction AUCs for different feature extraction methods and machine learning algorithms using T1CE+FLAIR: (A) prediction based on logistic regression; (B) prediction based on neural network; (C) prediction based on random forest; (D) prediction based on TPOT.

Figure 4 shows the predictive performance of the external dataset over MGMT promoter methylation status with T1CE and FLAIR images. Although there is a performance drop compared to internal dataset (AUC = 0.64 compared to AUC = 0.76), spherical radiomics continued to outperform conventional Euclidean radiomics by approximately 8%. Moreover, across different image modalities, combining all modalities yields consistently higher AUC than any sub-combinations, as shown in Supplementary E.

### Clustering Comparison between Spherical and Euclidean Radiomics

**Figure 5.**
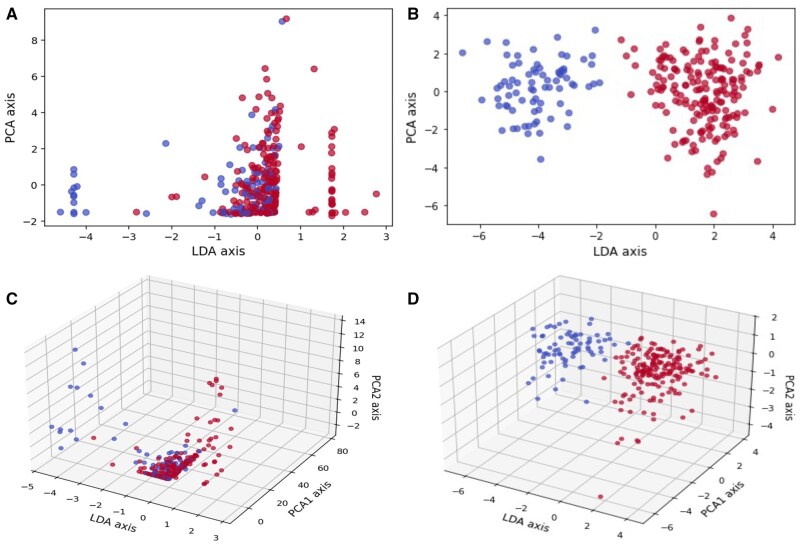
MGMT label supervised clustering with LDA and PCA projection on GLCM-typed features (blue: patient with negative MGMT label, red: patient with positive MGMT label): (A) 2D space clustering with Euclidean 2D radiomics. (B) 2D space clustering with Spherical radiomics. (C) 3D space clustering with Euclidean 2D radiomics. (D) 3D space clustering with Spherical radiomics.

The benefits of performing radiomic extraction in spherical rather than Euclidean space were further assessed using clustering analysis. To align with our top-performing predictive models (LR and NNs), we employed principal component analysis (PCA) for dimensionality reduction, taking advantage of its linear projection properties. Figure 5A-D illustrates an example in which GLCM-derived features were clustered in 2D and 3D spaces using linear discriminant analysis (LDA) applied to the PCA projections, comparing features extracted from Euclidean and spherical surfaces. The results demonstrate that GLCM features from spherical radiomics exhibit markedly clearer separation between clusters in both 2D and 3D visualizations compared with their Euclidean counterparts. For instance, in the 2D space, spherical radiomics yielded well-defined clusters, whereas Euclidean radiomics displayed only partial clustering at the extremes, with a large mixed region persisting in the center.

### Interpret GBM Biological Heterogeneity with Spherical Radiomics

Single-cell and spatial transcriptomic studies indicate that GBM exhibits 5 cellular layers radiating from the necrotic core to infiltrated normal brain,^24^ as illustrated in Supplementary F. We evaluated 93 radiomic features across 4 MRI-defined regions. Statistical comparisons between regions were performed using t-tests or Mann-Whitney U tests, depending on normality assessed by the Shapiro-Wilk test. To account for multiple comparisons, *P* value were adjusted using the Benjamini-Hochberg false discovery rate (FDR) correction,^39^ and statistical significance was defined as FDR-adjusted *P* < .05. As shown in Supplement Table S3, the largest differences were observed between the T2 hyperintense region and the 2-cm peritumoral extension zone, while differences between the necrotic core and enhancing T1 region were less pronounced.

These results reveal that GBM radiomics exhibit both a radial transition among different tumor sub-volumes and intra-spherical layer heterogeneity. A steeper radial transition in MGMT-unmethylated tumors (negative) was observed compared to MGMT-methylated tumors in T1CE images. Such trends would have been masked in Euclidean radiomics.

### Tumor Heterogeneity Described by Spherical Radiomics

Fitting the double sigmoid function to various radiomic features revealed that it effectively characterizes 2 distinct transitions in feature intensity as a function of radial distance from the tumor center. The first transition typically occurred at the boundary between the necrotic and contrast-enhancing tumor core (T1) and the adjacent T2/FLAIR hyperintense region, reflecting a shift from dense tumor cellularity and necrosis to infiltrative edema. The second transition was observed near the periphery of the T2 abnormality, extending into the clinically defined 2-cm expansion zone, and corresponded to an additional change in radiomic intensity and heterogeneity. Although often radiographically subtle, this region is known to harbor infiltrative tumor cells and is routinely encompassed within surgical or radiation treatment margins. Figure 5 illustrates an example of a double sigmoid fit for the first-order mean feature in patients stratified by MGMT promoter methylation status, and the corresponding fitting parameters and qualities across the whole patient cohort are summarized in Supplement Table S4.

**Figure 6.**
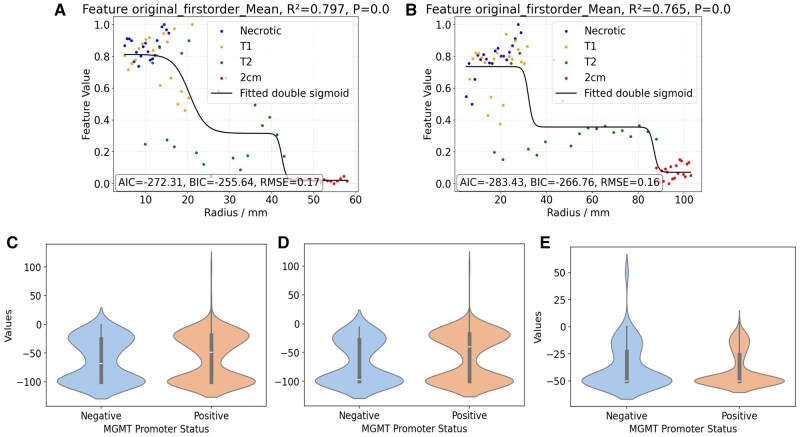
Double sigmoid fit for first-order mean feature in T1CE: (A) A patient with negative MGMT methylation; (B) A patient with positive MGMT methylation. Radiomic feature values in different regions are represented in different colors (blue, yellow, green, and red). $R^{2}$ represents the coefficient of determination and measures the percentage of variance in the data explained by the model. *P* value measures the statistical significance of the fitted relationship, where *P* < .05 means the fitted relationship is highly statistically significant. Violin plot of the transition slope k1 of first-order mean feature between negative MGMT promoter methylation (blue) and positive MGMT promoter methylation (orange) for different modalities (Mann-Whitney U test): (C) T1CE (*P* = 0.42); (D) FLAIR (*P* = 0.04); (E) ADC (*P* = 0.87). The central white in the black color bar represents the median of the data, and the shape shows the probability density of the data.

We performed univariate statistical analysis using the Mann-Whitney U test to evaluate the discriminative power of seven radiomic fitting parameters with respect to MGMT promoter methylation, EGFR and PTEN mutations, as well as survival status across different radiomic features. Following FDR correction (*P* < .05), approximately 6% of parameters demonstrated significant associations with MGMT promoter methylation, while 15% were significantly associated with EGFR mutation. Among these, the slope parameters k1 and k2, which characterize the steepness of radiomic transitions across regions, accounted for 21% of MGMT-associated features and 47% of EGFR-associated features. Notably, 71% of the significant slope parameters were k1, underscoring its potential sensitivity to genomic status. These results suggest that spatial radiomic transitions—particularly those quantified by k1—may capture underlying molecular heterogeneity and hold promise as imaging biomarkers. Figure 6A and B illustrate the differences in transition slope k1 between MGMT promoter methylation groups for the first-order mean feature, while Figure 6C and E visualizes an example of the distinct k1 values observed in 2 representative patients with differing MGMT promoter methylation status.

### Feature Significance Analysis

To interpret the trained models and identify the most predictive radiomic biomarkers, we employed SHapley Additive exPlanations (SHAP),^40^ which quantifies the contribution of each input feature to model predictions at the individual case level and enables ranking of features by overall importance. For illustration, we examined models predicting MGMT promoter methylation and EGFR mutation status shown in Supplementary G. The top 10 most influential features for MGMT and EGFR predictions are shown in Figure S13(I) and (II), while the top features for PTEN and survival predictions are presented in Figure S14(I) and (II). Among feature categories, gray-level co-occurrence matrix (GLCM) features were particularly prominent, consistently surpassing other categories. For instance, GLCM-based features achieved an AUC of 0.77 for MGMT and 0.68 for EGFR prediction (Figure S13(III)). Moreover, their relative representation increased when comparing the original feature pool to the selected significant features: for MGMT and EGFR, GLCM features rose from 25.6% of the original set to 33.4% and 37.2% of the selected features, respectively (Figure S13(IV)). Similar patterns were observed for PTEN and survival prediction (Figure S14(III) and (IV)). In addition to GLCM features, other categories—including first-order statistics and gray-level dependence matrix (GLDM) features—contributed meaningfully. As summarized in Figure S13(V) and Figure S14(V), these features achieved competitive AUCs and comprised a notable proportion of the significant predictors, highlighting their complementary predictive value. These results indicate that GLCM features drive model performance, while integrating multiple feature types enhances predictive characterization of molecular and survival outcomes.

## Discussion

The ability to non-invasively predict molecular markers such as MGMT, EGFR, and PTEN, as well as patient survival, has important clinical implications. For MGMT, accurate preoperative prediction of promoter methylation status could identify patients most likely to benefit from alkylating chemotherapy (eg, temozolomide). Similarly, prediction of EGFR alterations, including EGFRvIII, may guide enrollment in targeted therapy trials and provide prognostic information. PTEN, a critical tumor suppressor gene, has been linked to radiation sensitivity and resistance to anti-angiogenic therapies.^41^ Some studies have associated PTEN loss with poor survival,^42^ whereas others reported no significant correlation,^43^ underscoring the complexity of its role in GBM biology. Beyond molecular markers, accurate imaging-based prediction of survival status could provide clinicians with valuable insight into disease trajectory and support more personalized treatment planning.^22^ Early prediction of patient survival is critical to improve the balance between the aggressiveness of intervention and the patients’ quality of life.

In addition to their direct predictive value, imaging-based approaches complement biopsy-based testing, which is limited by sampling error, sparse time points, and intratumoral heterogeneity. Our spherical radiomics framework offers several further clinical advantages. First, by capturing distinct radiomic signatures across tumor compartments, this approach can support more personalized surgical planning by improving margin delineation that reflects true tumor infiltration. Second, its capacity to characterize peritumoral heterogeneity may help refine radiotherapy target volumes, enhancing local control while reducing exposure to healthy tissue. Finally, the interpretability and spatial structure of spherical radiomic features offer opportunities for integration into clinical decision-support tools, enabling more precise radiogenomic stratification and personalized treatment strategies.

Nevertheless, predicting genomic status directly from medical images remains challenging, with prior studies reporting modest and variable levels of performance. For example, Sasaki et al achieved a prediction accuracy of 0.67 for MGMT prediction,^44^ Haubold et al achieved an AUC of 0.74,^45^ and Xi et al reported an accuracy of 0.8,^46^ while the MICCAI 2021 challenge reported a best AUC of 0.62 for MGMT.^47^ Our observations using standard Euclidean-space radiomics achieved AUC ∼0.6, similar to the MICCAI 2021 challenge result. For EGFR prediction, Rathore et al reported a best AUC of 0.80 using the Hospital of the University of Pennsylvania dataset with Euclidean-space radiomics.^48^ Similarly, Kazerooni et al^49^ reported AUCs of 0.68 for EGFR and 0.73 for PTEN using conventional MRI, while Hu et al^13^ achieved accuracies of 0.75 and 0.69 for EGFR and PTEN, respectively, with 3D Euclidean-space radiomics. Beyond genomic prediction, radiomics has also been applied to survival modeling: Bae et al reported an AUC of 0.65 for survival status prediction using Euclidean-space radiomics,^50^ and Shboul et al^51^ achieved an accuracy of 0.68 when employing a radiomics feature-guided NN. It is worth noting that the aforementioned performances were achieved under varying experimental conditions, making direct comparison difficult. For instance, customized cutoff thresholds were used to boost performance, but such approaches substantially limit the generalizability of ML models across datasets from different institutions.^52^

When projecting spherical coordinates onto a 2D plane, a discontinuity is introduced at the angular boundary (eg, −*π* to + *π*). To assess the impact of this boundary effect, we evaluated alternative angular parameterizations and observed consistent results, indicating that the framework is robust to the choice of angular cut. Future work may incorporate periodic boundary conditions to further address angular continuity. Regarding Euclidean-space radiomics, although 3D radiomics is often assumed to better capture tumor heterogeneity, prior studies have reported inconsistent findings, with some showing comparable or even inferior performance relative to 2D approaches.^53^ This variability may be attributed to differences in feature extraction, dimensionality, noise sensitivity, and model selection.

In this study, we demonstrated that spherical radiomics achieves statistically significant and substantially higher prediction accuracy than Euclidean space radiomics for genomic features (EGFR, MGMT, PTEN) and survival *under identical training and testing conditions*. To understand what contributes to the improved prediction performance, it is essential to discuss the foundation of radiomics. The values of radiomics features are driven by the voxel-level heterogeneity in the image intensity.^54^ Radiogenomics is based on the hypothesis that imaging features may reflect underlying genomic and microenvironmental characteristics of tumors, although such relationships are often indirect and not spatially resolved. However, despite the large panel of radiomics features that have been devised, they do not by default interpret the high-level and global architecture that is the hallmark of GBM.^24^ The current study manually encoded a simple tumor global architecture consistent with the native tumor development into the radiomics study. In other words, radiomics’ ability to perform quantitative imaging texture analysis needs to be augmented by structural decomposition of the tumor architecture. The performance of radiomics thus depends on the methods of decomposition. The observation is further substantiated by the clustering analysis that spherical radiomics features are more distinctly separated by the biomarker and survival statuses. The transition of the radiomics values also follows the intrinsic radial structure of the GBM, which would have been missed by the standard radiomics analysis performed on a Euclidean grid. Interestingly, the gradient of transition appears to be associated with GBM biomarkers such as MGMT methylation status; however, the biological interpretation of this relationship remains indirect and requires further validation with spatially resolved molecular or histopathological data.

The comparative evaluation of predictive models demonstrated an overall advantage of the NN relative to the baseline approaches. Compared with image-based deep learning frameworks such as convolutional neural networks, the NN-based radiomics approach offers notable advantages. By leveraging handcrafted features extracted via radiomics, it bypasses the need for large-scale training datasets while still achieving competitive or superior predictive accuracy. This efficiency makes it especially suitable for studies with limited cohort sizes, such as those commonly encountered in GBM research.

Nonetheless, LR remains widely adopted in radiogenomic studies due to its interpretability and robustness.^55–57^ In our study, although NN achieved marginally higher predictive accuracy, the current sample size and variability limit definitive conclusions about its advantage over LR, warranting validation in larger cohorts. Beyond the comparison of prediction models, this study also emphasizes the added value of integrating spherical radiomic features derived from multiple imaging modalities to enhance predictive accuracy. By leveraging complementary information across modalities, spherical multimodal analysis captures distinct but interrelated aspects of tumor biology, including ADC, T1CE, and FLAIR. This integrative strategy provides a more comprehensive characterization of tumor phenotype than any single modality. Our results demonstrate that combining multimodality input with spherical spatially resolved features yields further improvements, suggesting that both the choice of feature representation and the integration of complementary imaging data are crucial for advancing radiogenomic prediction.

The study has several limitations that we wish to discuss here.

1. Generalizability: The study was performed on data from a single institution and validated on another institution only regarding MGMT prediction. The performance drop between internal and external data suggests that robustness and generalizability will require future studies pending the availability of more matching external data, including the same imaging sequences, biomarkers, and clinical information.
2. Feature extraction: GBM infiltration is known to be anisotropic; the current framework focuses on quantifying the macroscopic spatial gradients in imaging phenotypes relative to the tumor centroid. Future work would integrate anisotropic spatial descriptors, such as higher-order spherical harmonics, to refine the current representation. Besides, due to the thin-shell design (∼1 voxel thickness), radiomic features were extracted from shell surfaces rather than volumes. Future work will explore extensions incorporating 3D and directional heterogeneity.
3. Feature stability and biological interpretation: Spherical radiomics offers a novel approach to feature extraction by capturing peritumoral patterns that may reflect underlying biological processes. However, the spherical representation is nonetheless a simplification of the complex tumor microenvironment, particularly in GBM, where diffusion, perfusion, and infiltration patterns are highly heterogeneous and anisotropic. Although spherical radiomics shows a significantly better correlation with several key GBM biomarkers and patient survival, the mechanistic understanding of the correlation remains challenging. The limitation also opens the door to future investigation of Radiomics in the full non-Euclidean space. Besides, future studies are needed to assess the sensitivity to spherical radiomics parameters across different patient cohorts (shell number, centroid definition, interpolation method, segmentation variability).
4. A major motivation of the current study came from spatially encoded transcriptomics. However, such information is unavailable for the current study patient cohort. Therefore, the spherical radiomics features were only correlated with biomarkers that are not specific to the tumor’s geometric location. A more in-depth analysis based on spatially encoded transcriptomic samples registered to multi-parametric MR images will likely yield additional insights into spherical radiogenomics.

## Conclusions

In this study, we developed a novel spherical radiomic framework for predicting MGMT promoter methylation, EGFR mutation, PTEN mutation, and survival status in GBM, integrating features derived from multiple imaging modalities. Compared with conventional 2D and 3D radiomics performed in the Euclidean space, spherical radiomics achieves consistently higher predictive accuracy across all modeling approaches. The inclusion of shape features further enhanced performance, highlighting the importance of spatial context in radiogenomic analysis.

## Supplementary Material

vdag132_Supplementary_Data

## Data Availability

The entire framework can be found on our GitHub page: https://github.com/Isaac0047/Shell_Radiomics.git. The raw data required to reproduce the findings presented in the paper are available to download from https://www.cancerimagingarchive.net/collection/ucsf-pdgm/. Requests for further information and resources should be directed to and will be fulfilled by the corresponding author, Ke Sheng (Ke.Sheng@ucsf.edu).
