# Supplementary material for: Spherical radiomics for radiogenomic assessment of glioblastoma heterogeneity
Source: Neurooncol Adv. 2026 May 16;8(1):vdag132. doi: 10.1093/noajnl/vdag132 (PMC13228145; doi:10.1093/noajnl/vdag132)
Supplement: vdag132_Supplementary_Data [file vdag132_supplementary_data.docx]

# SUPPLEMENTAL INFORMATION

# *A* Patient demographic comparison

Table S1 Patients demographic comparison of UCSF-PDGM and UPENN-GBM dataset

|  | UCSF-PDGM | UPENN-GBM |
| --- | --- | --- |
| No. of valid GBM patients | 299 | 87 |
| No. of men | 188 (63%) | 62 (71%) |
| No. of women | 111 (37%) | 25 (29%) |
| Mean age (y) | 62 ± 13 | 62 ± 11 |
| MGMT methylated | 214 (72%) | 37 (43%) |
| EGFR mutated | 129 (43%) | N/A |
| PTEN mutated | 169 (57%) | N/A |
| Survived (15-months) | 54 (21%) | N/A |

# *B* ROC curve for different folds


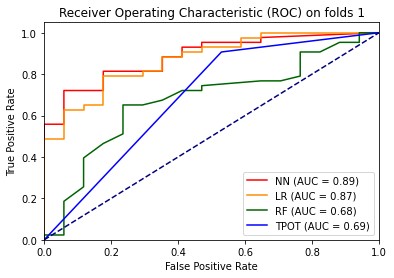

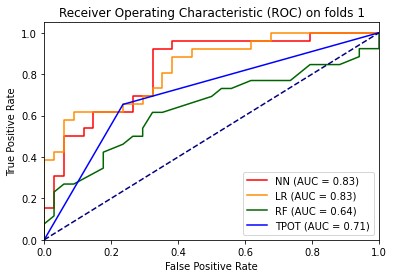

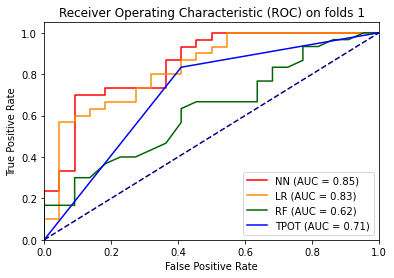

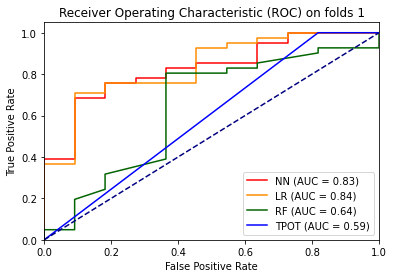


a

b

c

d

*Figure S1: ROC curves for different machine learning algorithms regarding (X-axis is False Positive Rate and Y-axis True Positive Rate) (a) MGMT prediction; (b) EGFR prediction; (c) PTEN prediction; (d) Survival status prediction.*

# *C* Shell contour mappings


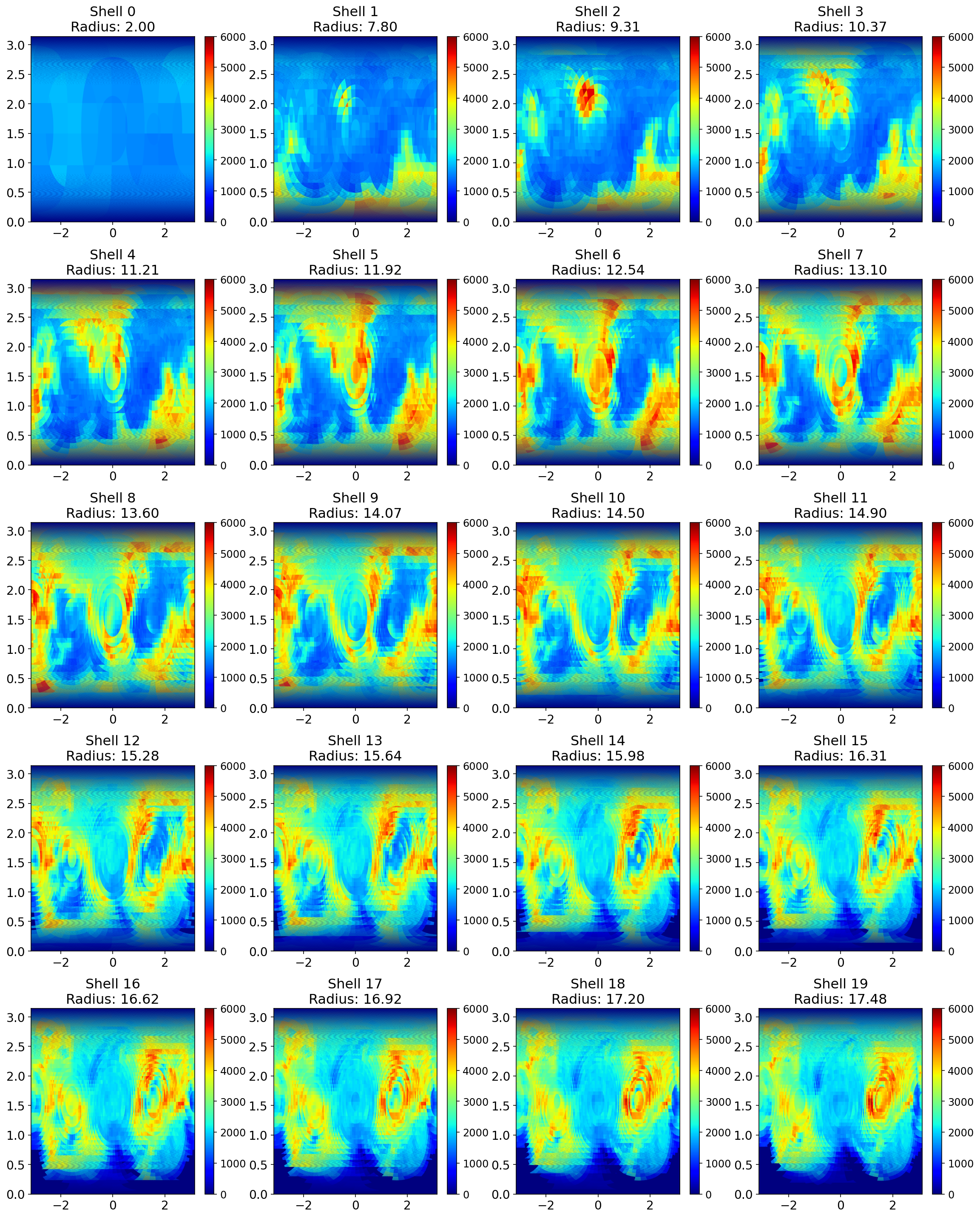


*Figure S2: Shell contour mapping for T1CE in the necrotic region*


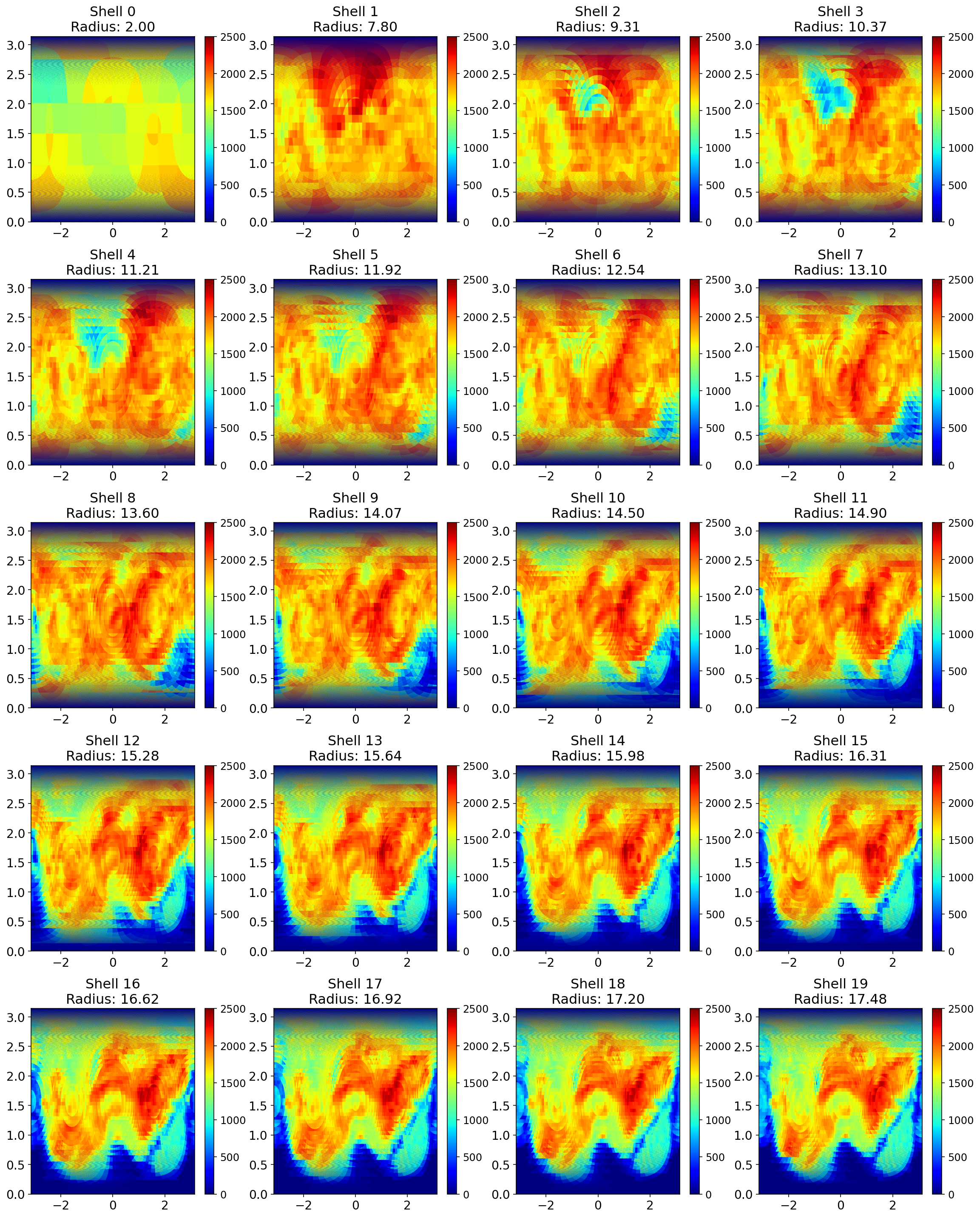


*Figure S3: Shell contour mapping for FLAIR in necrotic region*


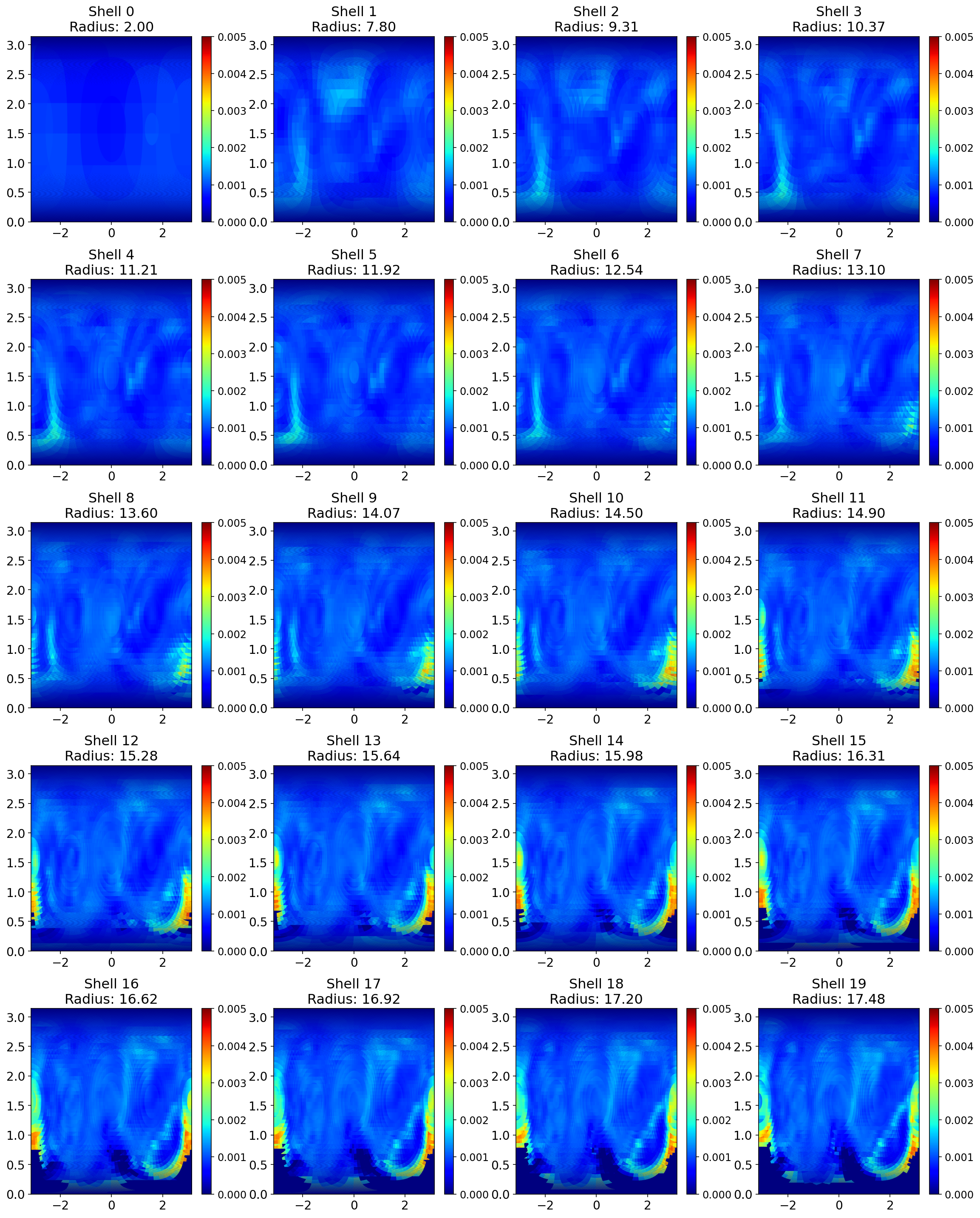


*Figure S4: Shell contour mapping for ADC in necrotic region*


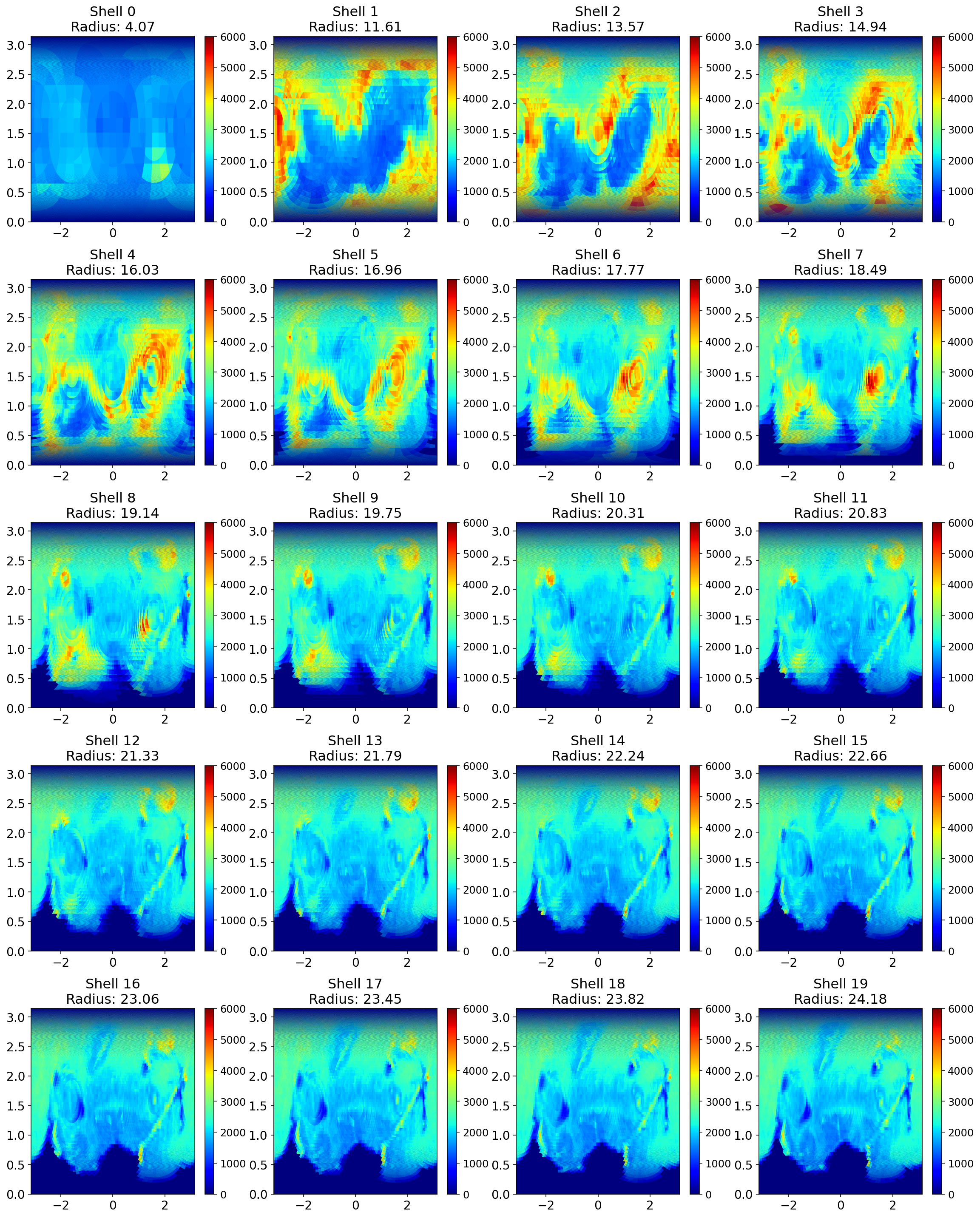


*Figure S5: Shell contour mapping for T1CE in T1 enhancing region*


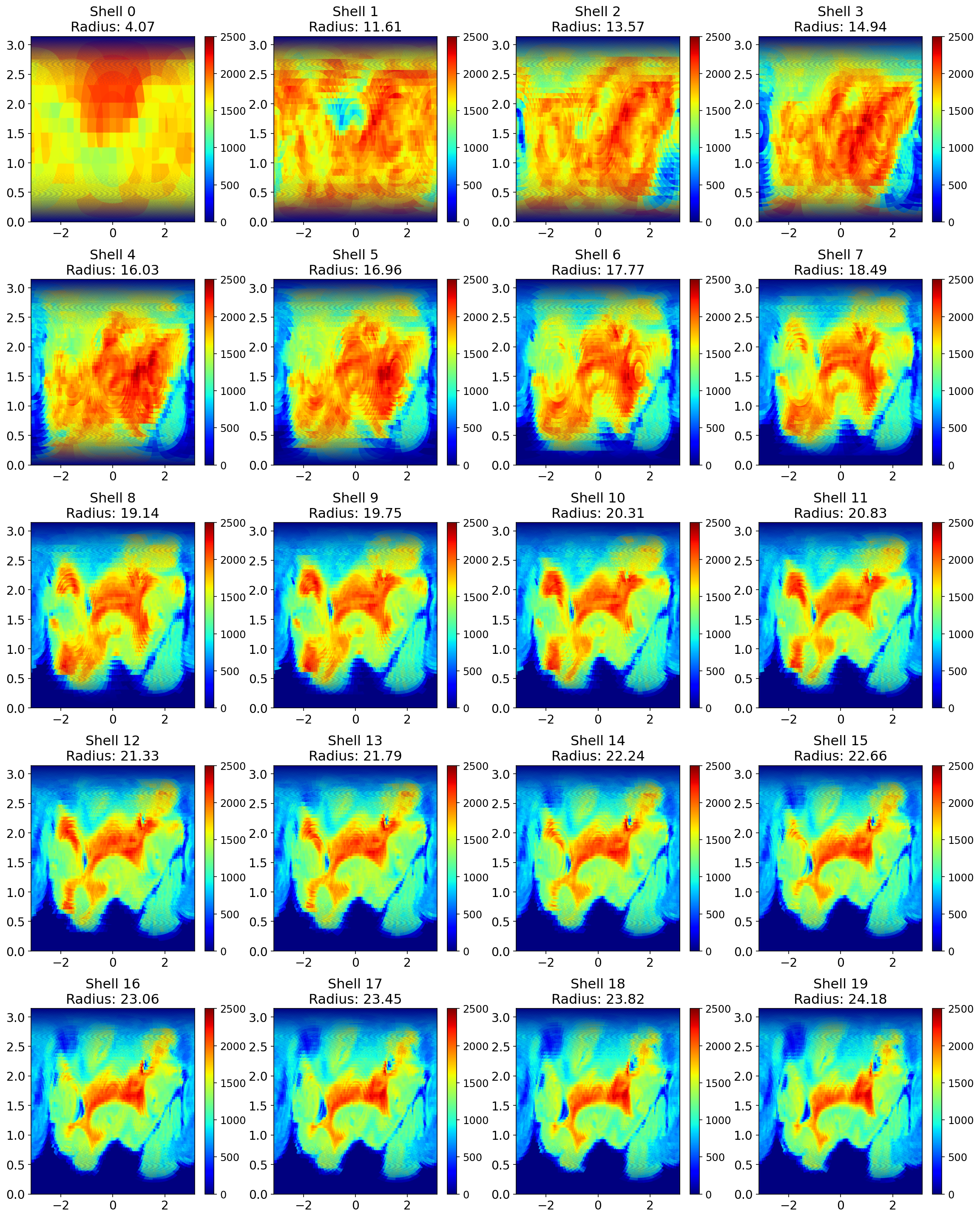


*Figure S6: Shell contour mapping for FLAIR in T1 enhancing region*


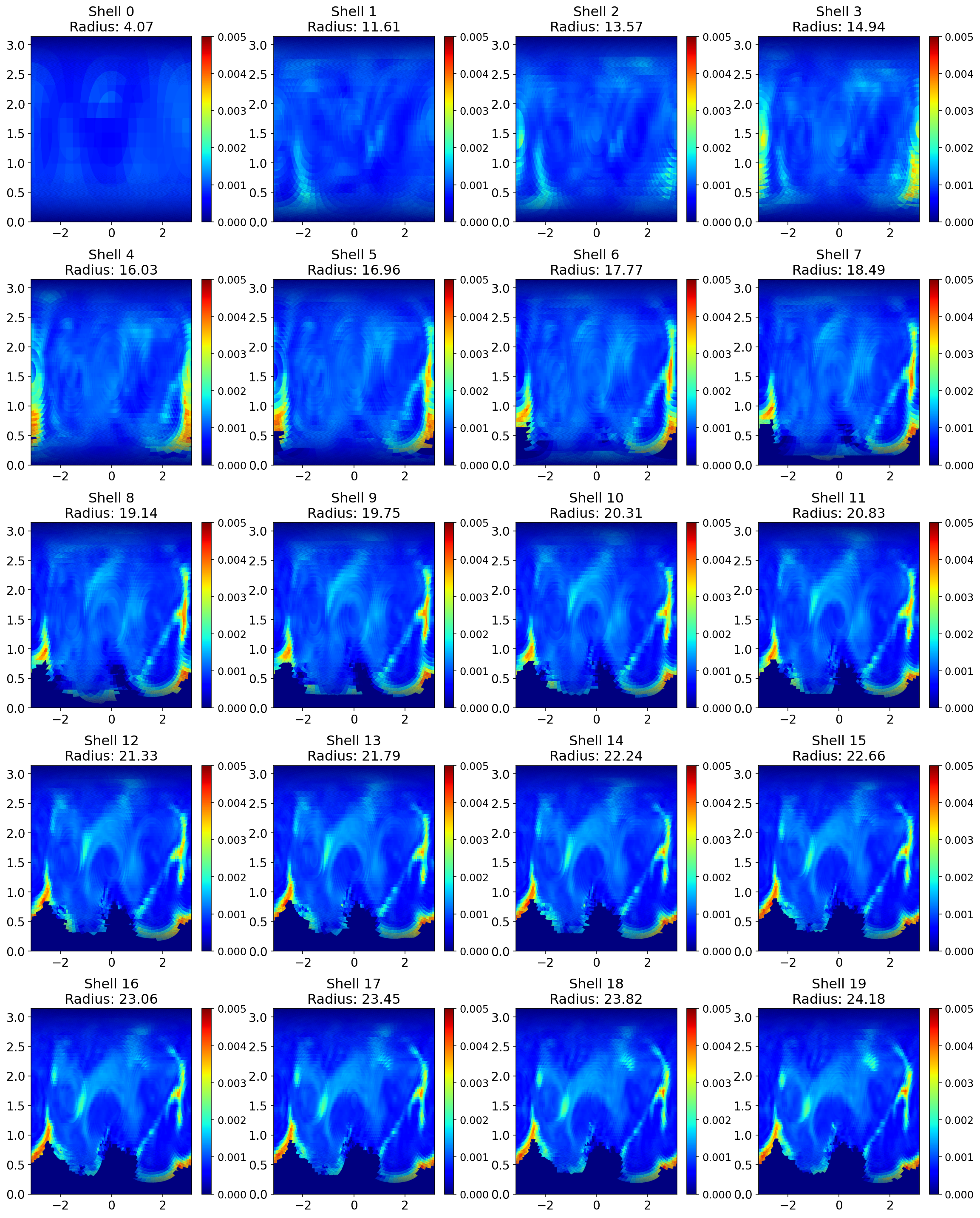


*Figure S7: Shell contour mapping for ADC in T1 enhancing region*


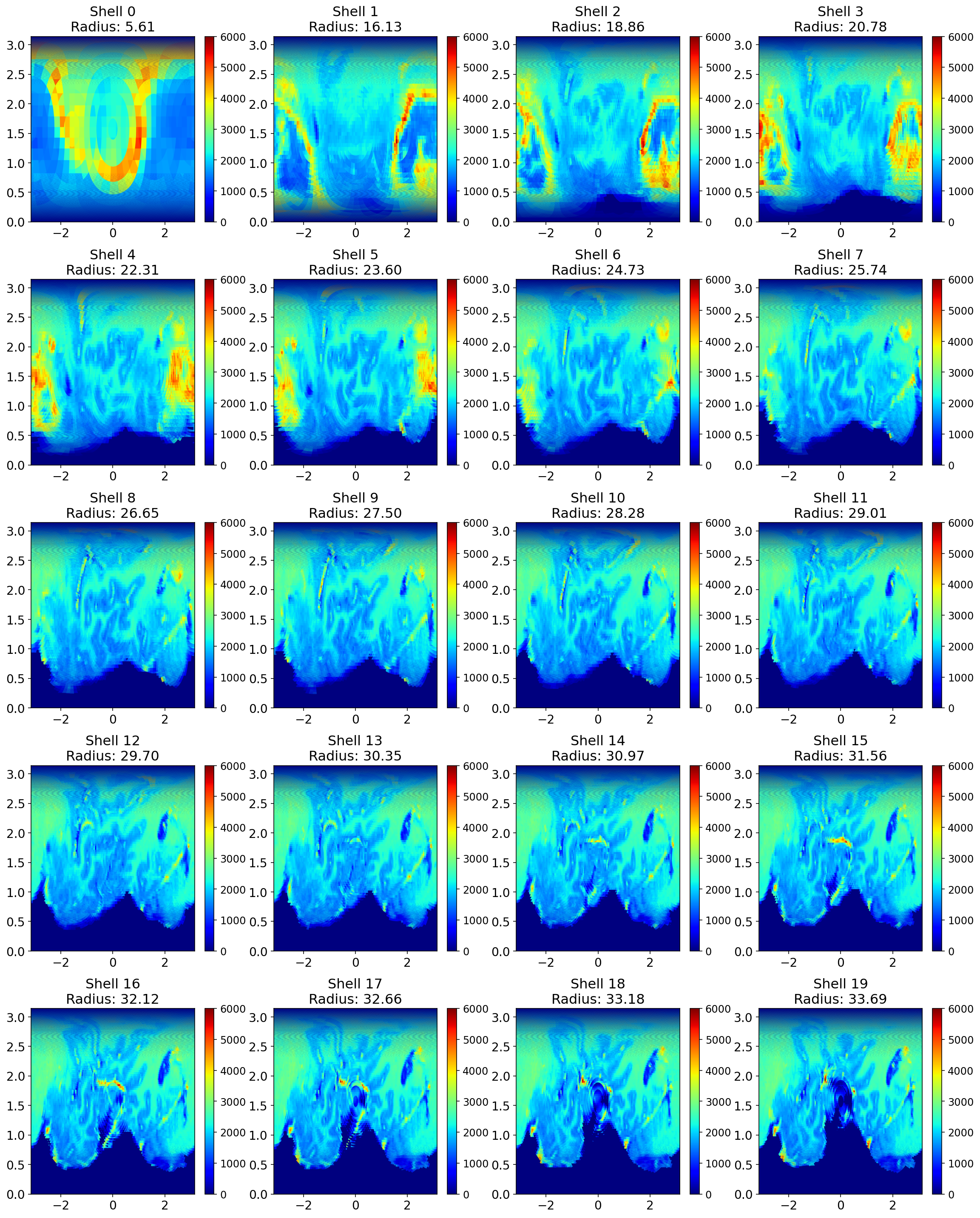


*Figure S8: Shell contour mapping for T1CE in T2 lesion region*


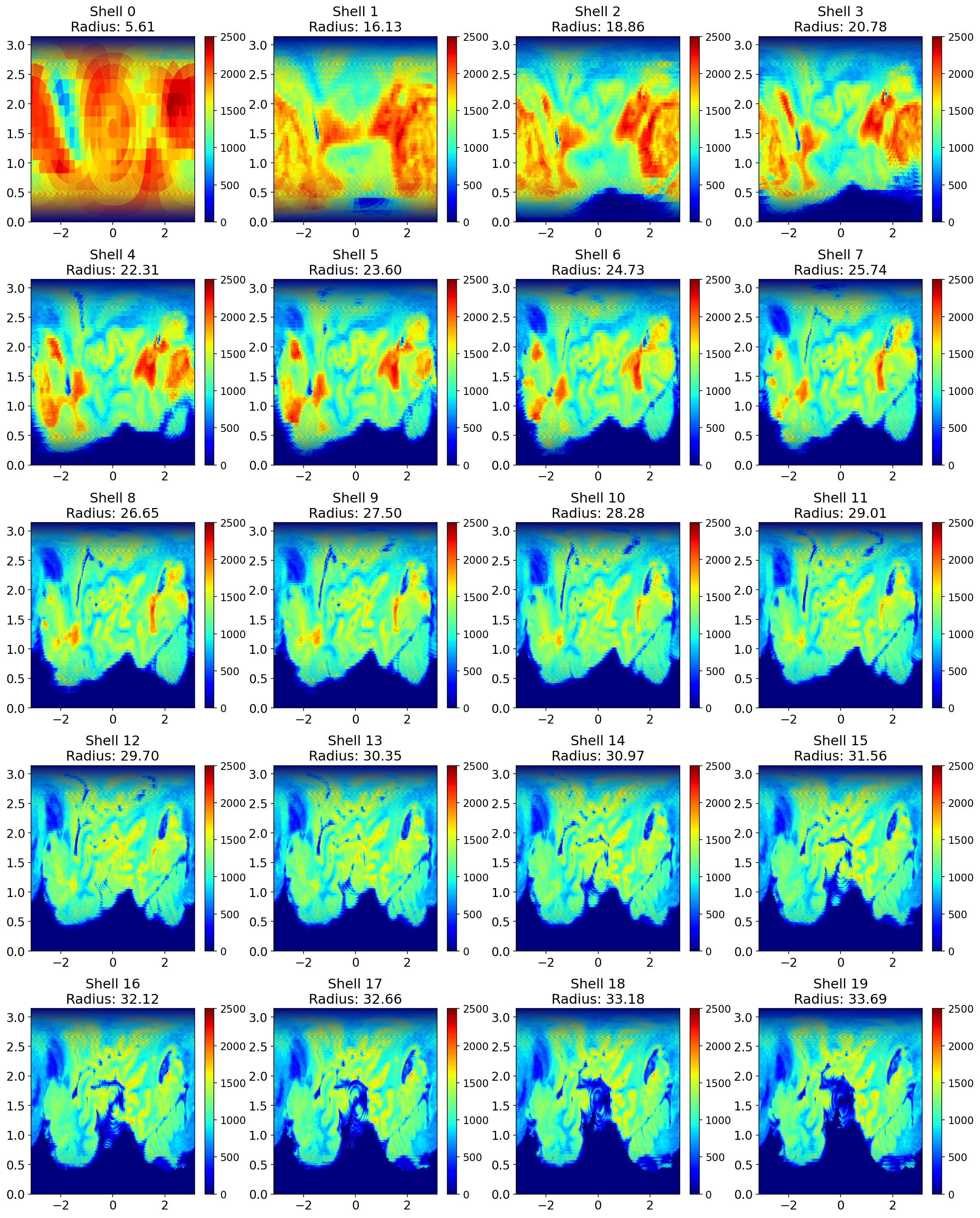


*Figure S9: Shell contour mapping for FLAIR in T2 lesion region*


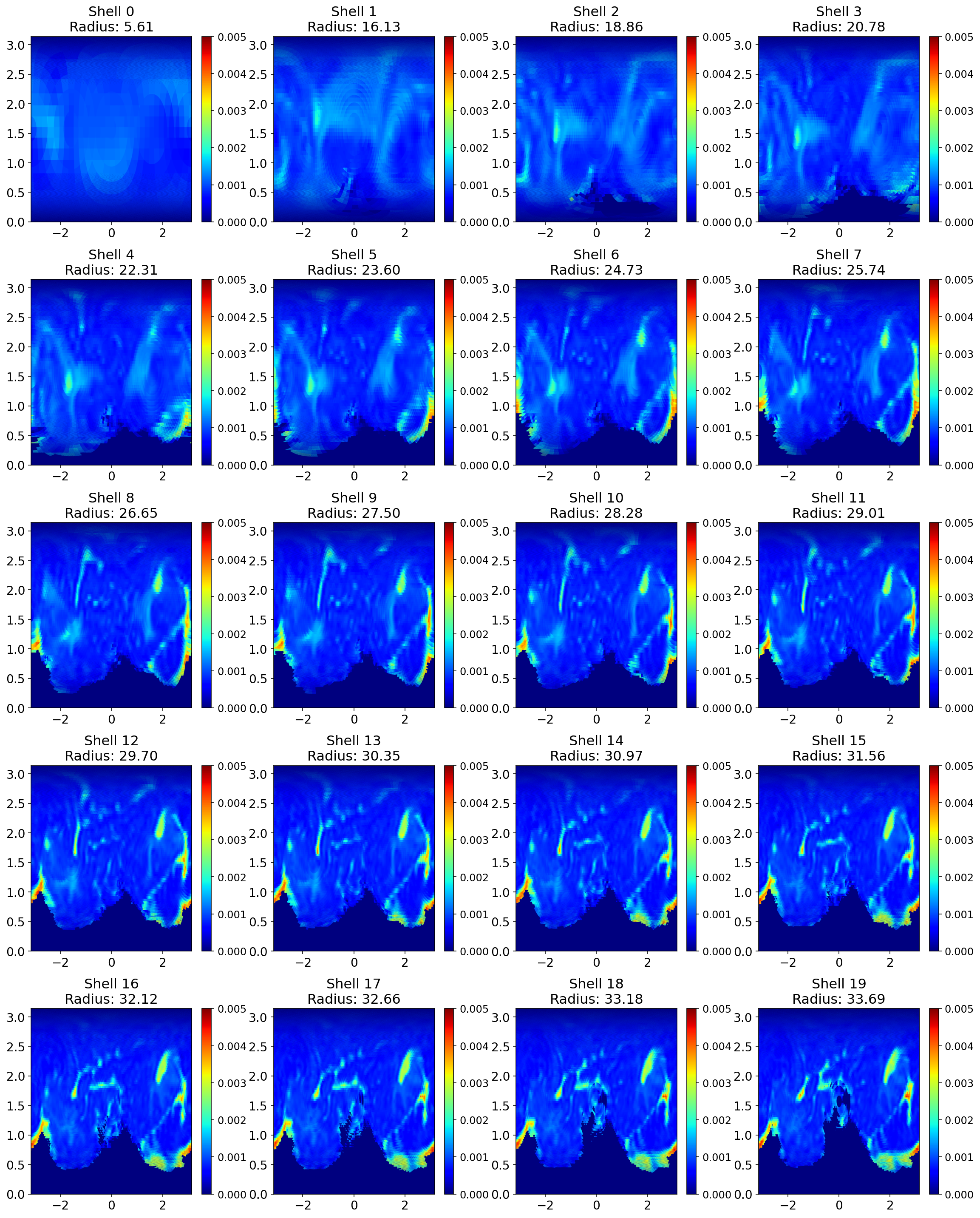


*Figure S10: Shell contour mapping for ADC in T2 lesion region*

## D FEATURE selection


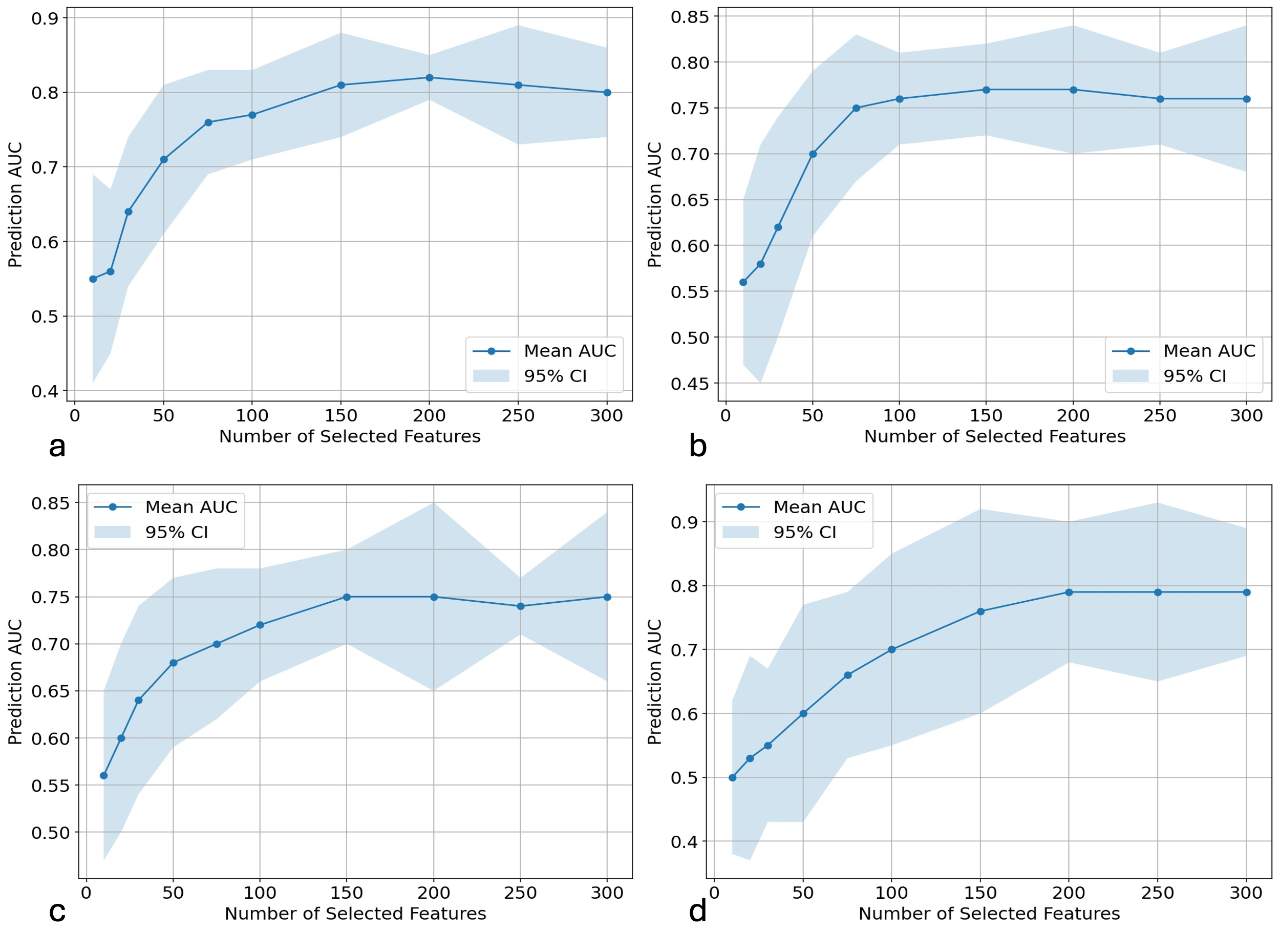


*Figure S11: Number of features’ influence on neural network’s prediction AUC (band denotes 95% confidence interval): (a) MGMT promoter methylation prediction AUC (b) EGFR mutation prediction AUC (c) PTEN mutation prediction AUC (d) Survival prediction AUC.*

## E Prediction accuracy across different algorithms and modalities based on spherical radiomics

a

b

c

d


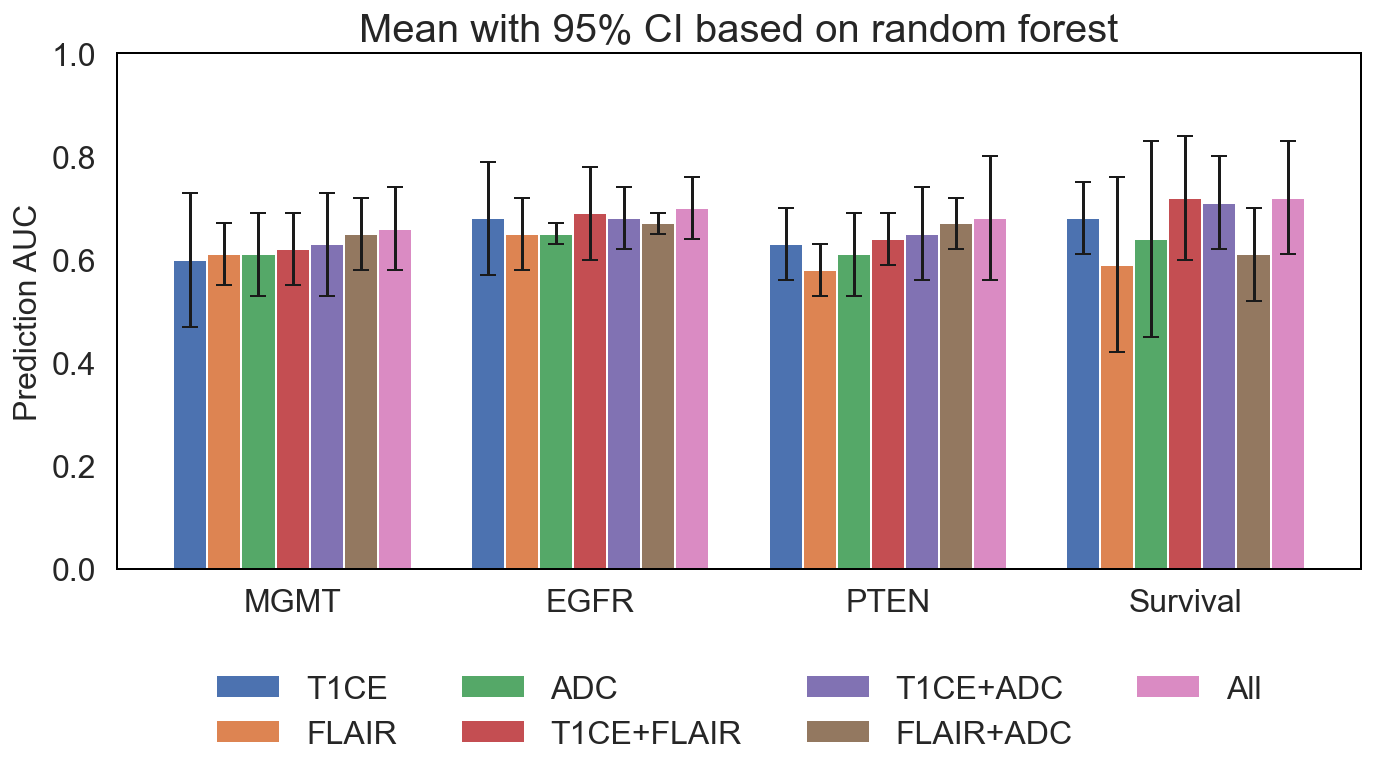

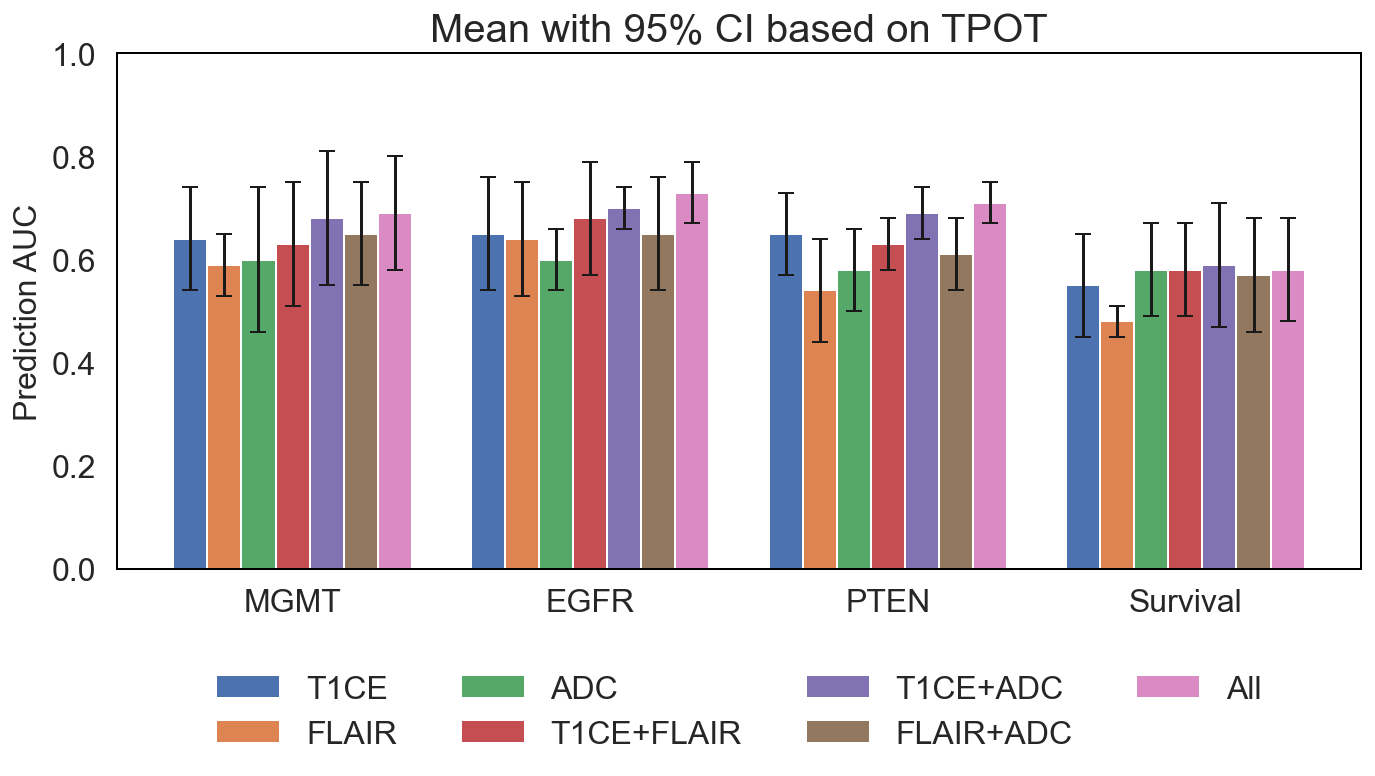

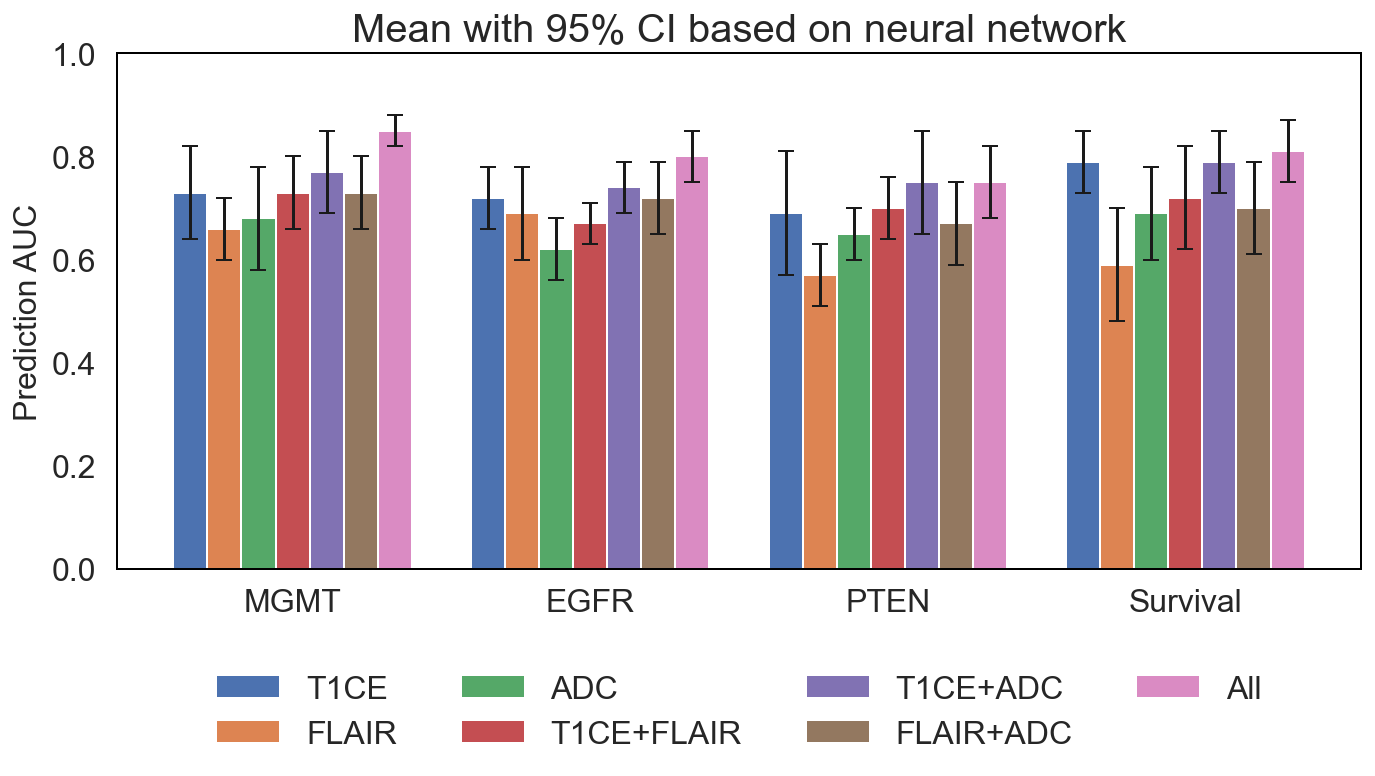

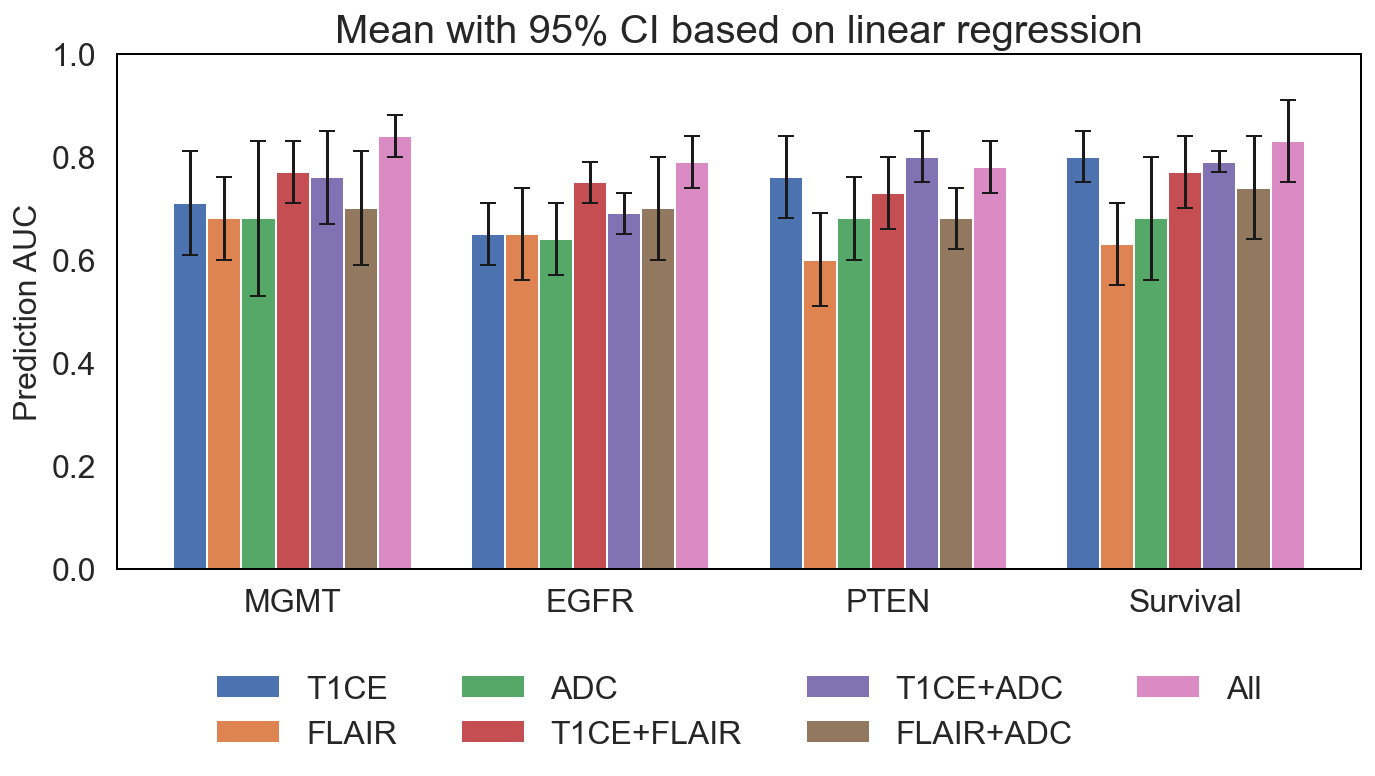


*Figure S12: Prediction AUCs for different modality combinations with different machine learning algorithms: (a) Prediction based on linear regression; (b) Prediction based on neural network; (c) Prediction based on random forest; (d) Prediction based on TPOT.*

## F Relationship between GBM layers and MRI zones

Table S2 Relationship between GBM layers and MRI zones

| GBM layers | Example Dominant Cell States | Functional Interpretation | GBM MRI Zone |
| --- | --- | --- | --- |
| Layer 1 | MES hypoxic/necrotic | Hypoxia/Necrotic Core | Necrotic core |
| Layer 2 | MES-like, MES-Ast | Hypoxia-associated States | T1-enhancing |
| Layer 3 | Angiogenetic, Vascular | Angiogenic Response and  Immune Hub | T1-enhancing |
| Layer 4 | Astrocyte-like,  Neural-progenitor-like | Malignant Neurodevelopment | T2 lesion |
| Layer 5 | Reactive Astrocytes, Neuron | Non-malignant Brain Cells | 2cm expansion, parenchyma |

## G identification of the most predictive radiomic biomarkers using SHAP


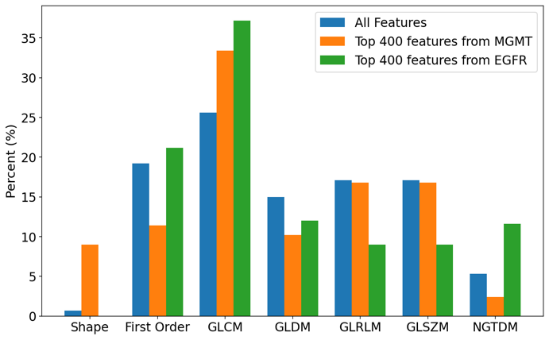


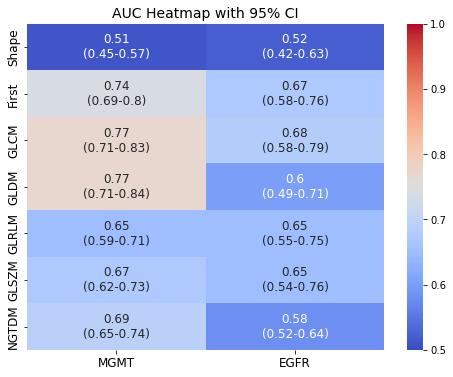


III. Prediction AUC with different radiomics

IV. Percentage of different radiomics


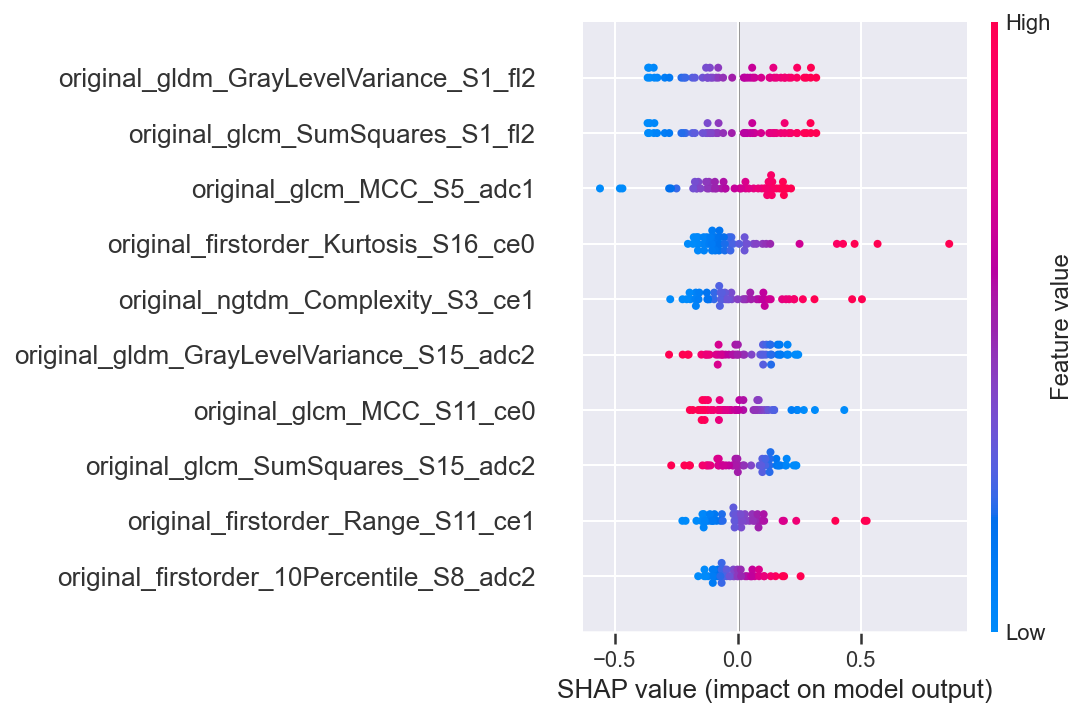

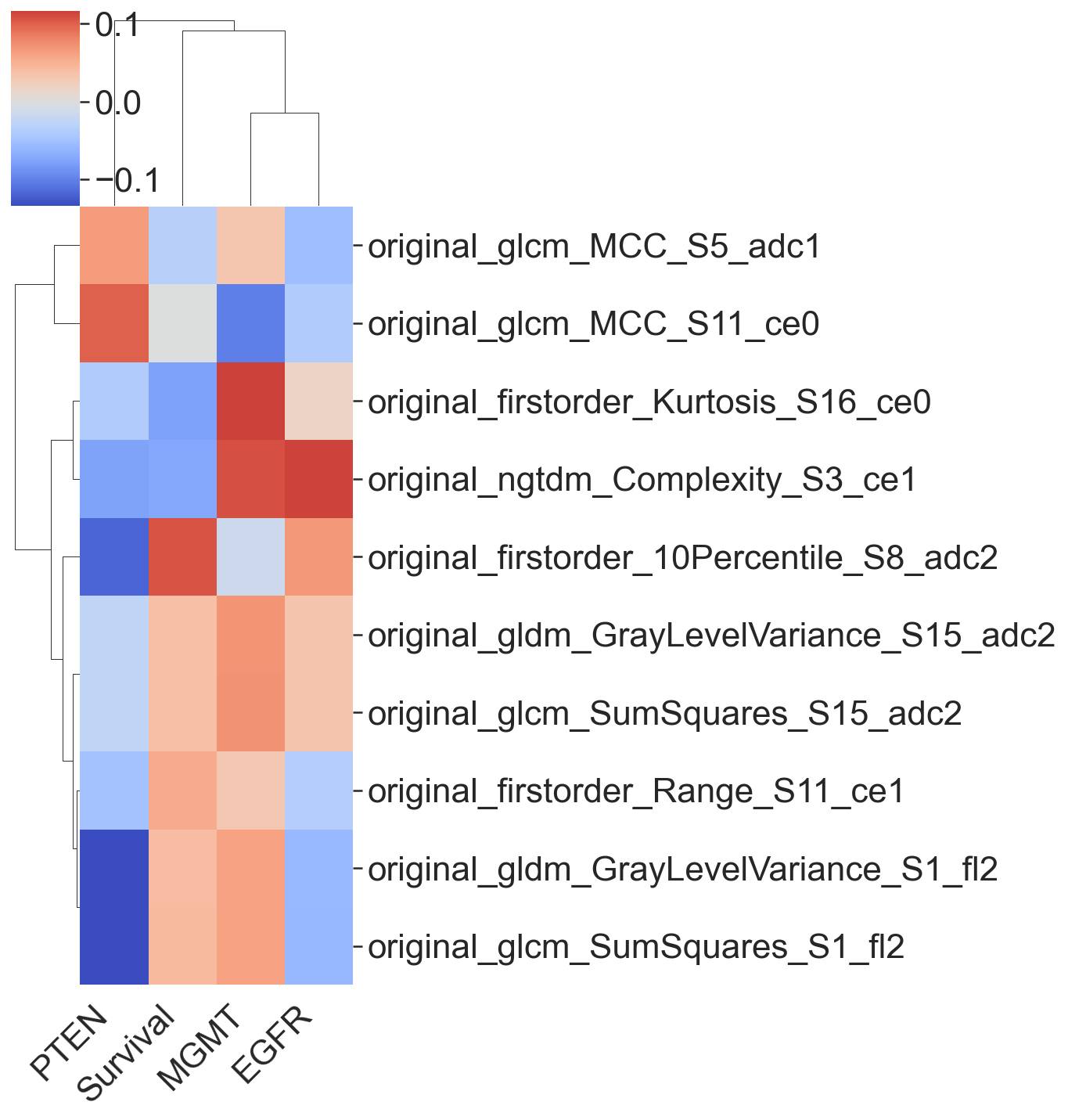


I. Top 10 radiomics for MGMT prediction


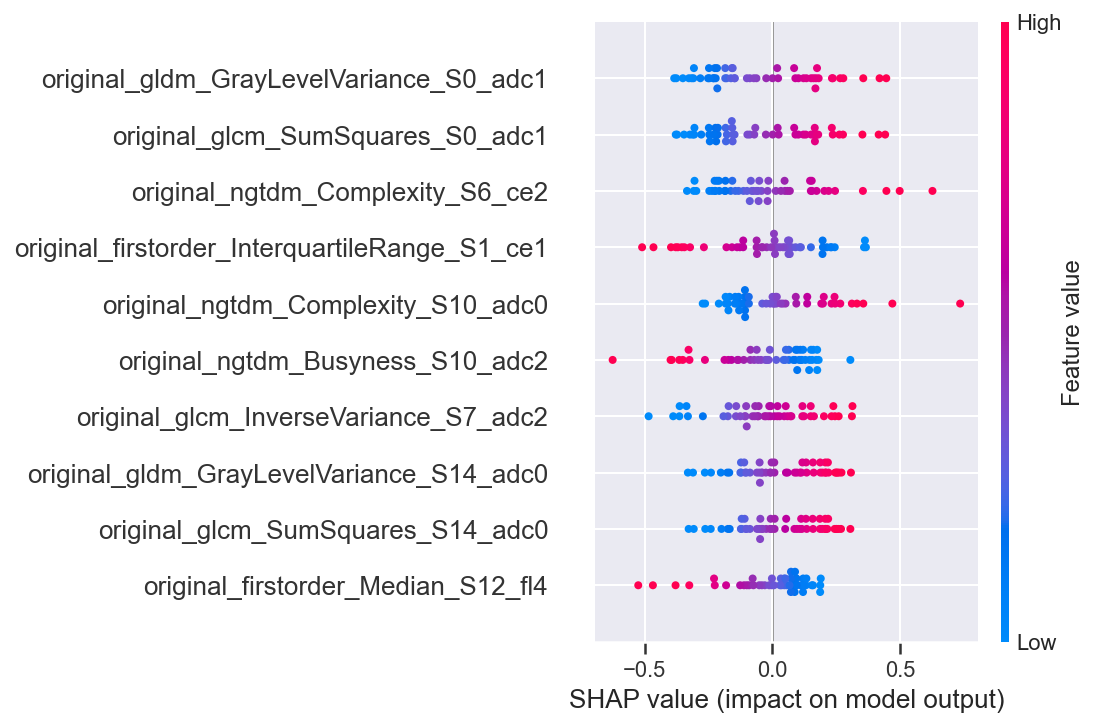

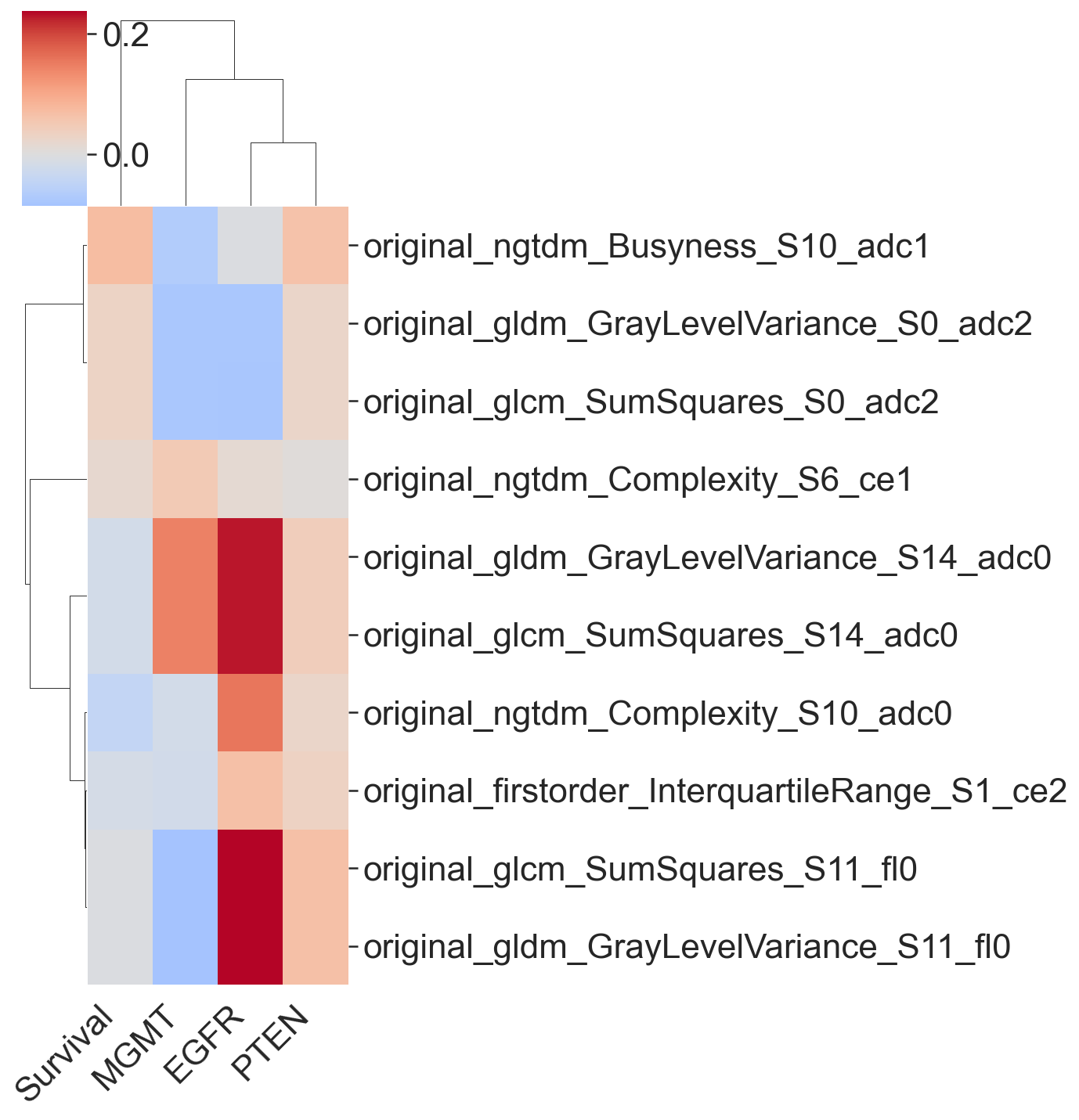


II. Top 10 radiomics for EGFR prediction

*Figure S13: Significant feature analysis: (I) SHAP analysis with hierarchical clustering for MGMT prediction; (II) SHAP analysis with hierarchical clustering for EGFR prediction; (III) Heatmap of prediction AUC across different radiomic feature categories; (IV) Distribution of radiomic feature types among all features (blue), the top 400 features ranked by MGMT prediction (orange), and the top 400 features ranked by EGFR prediction (green).*

*
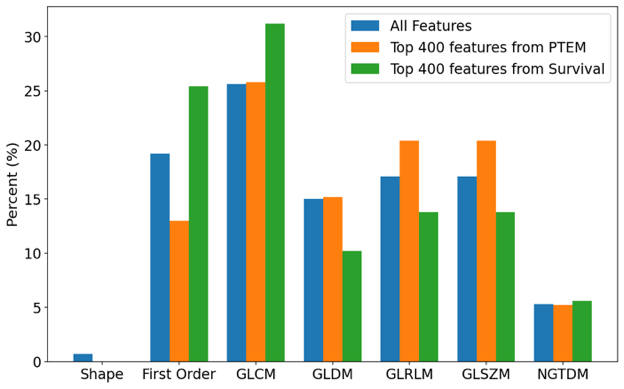
*


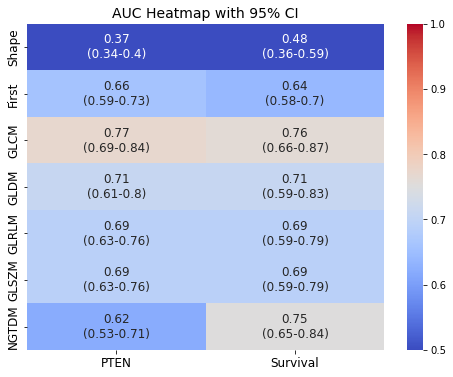

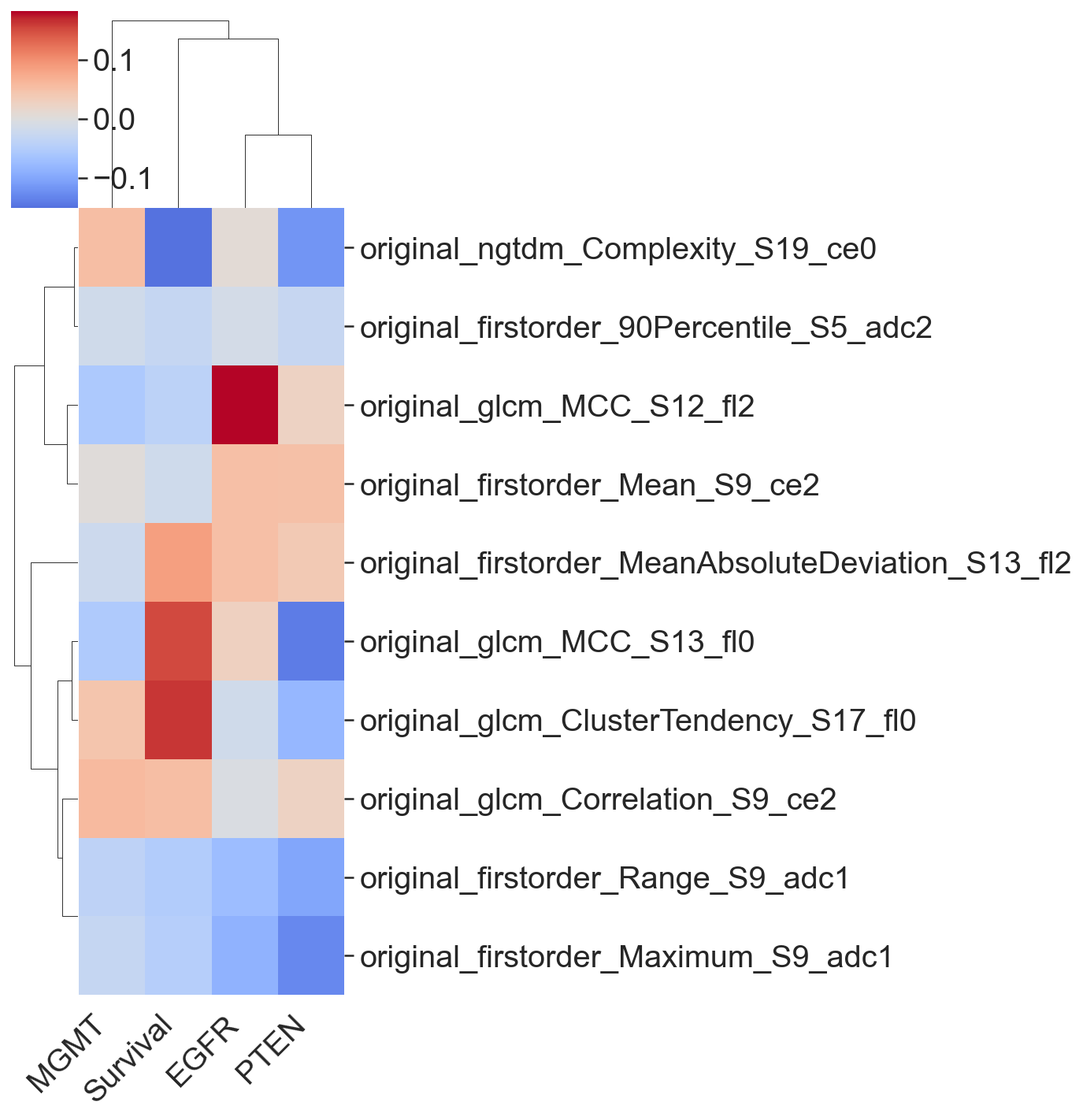

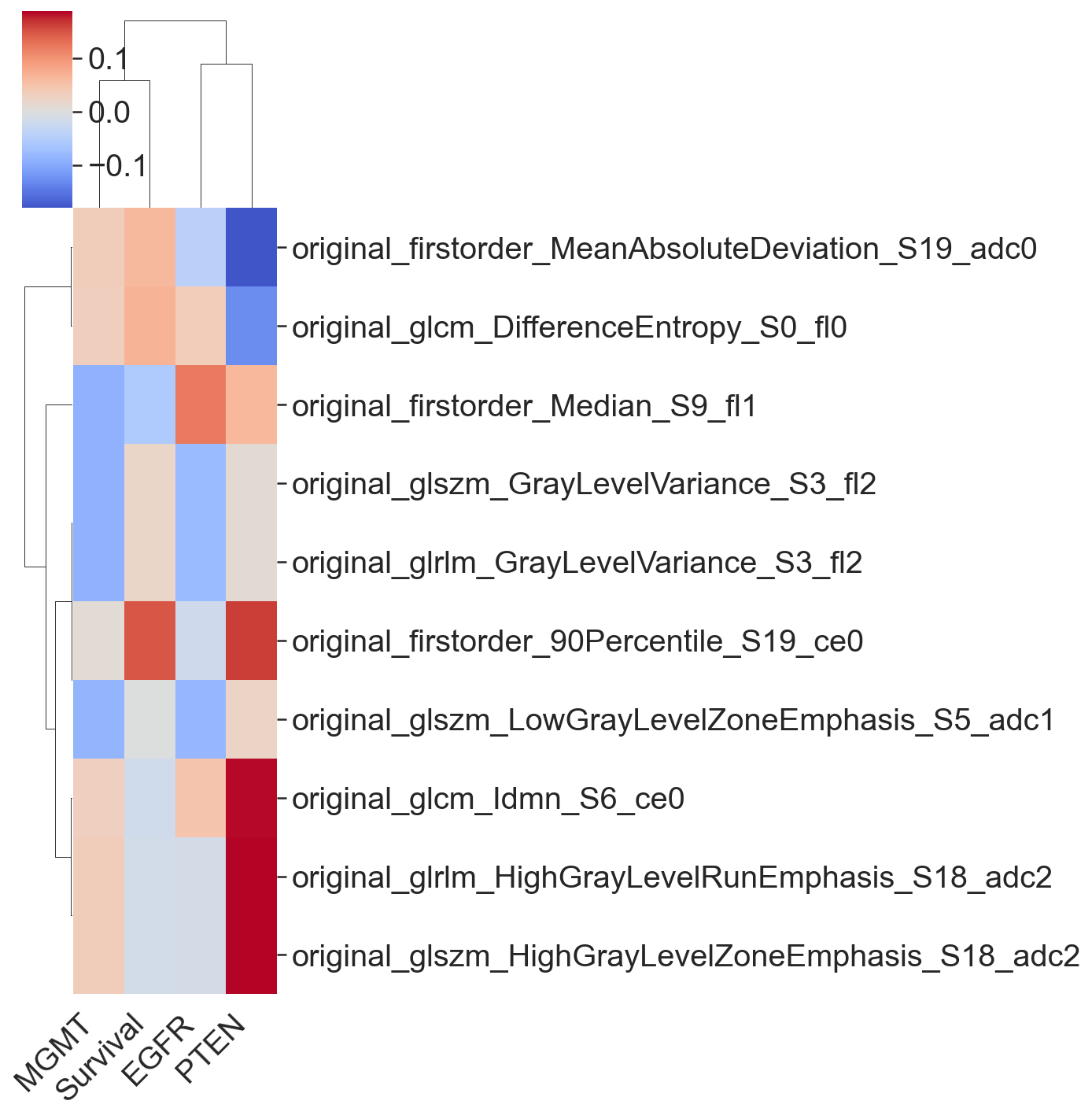

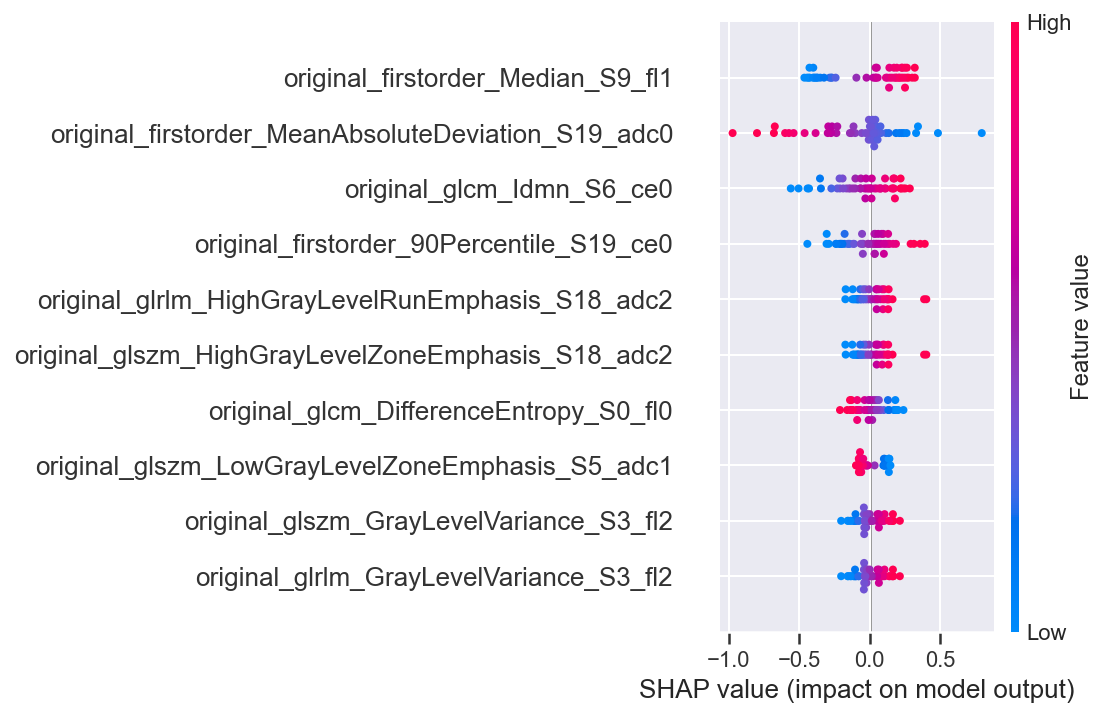

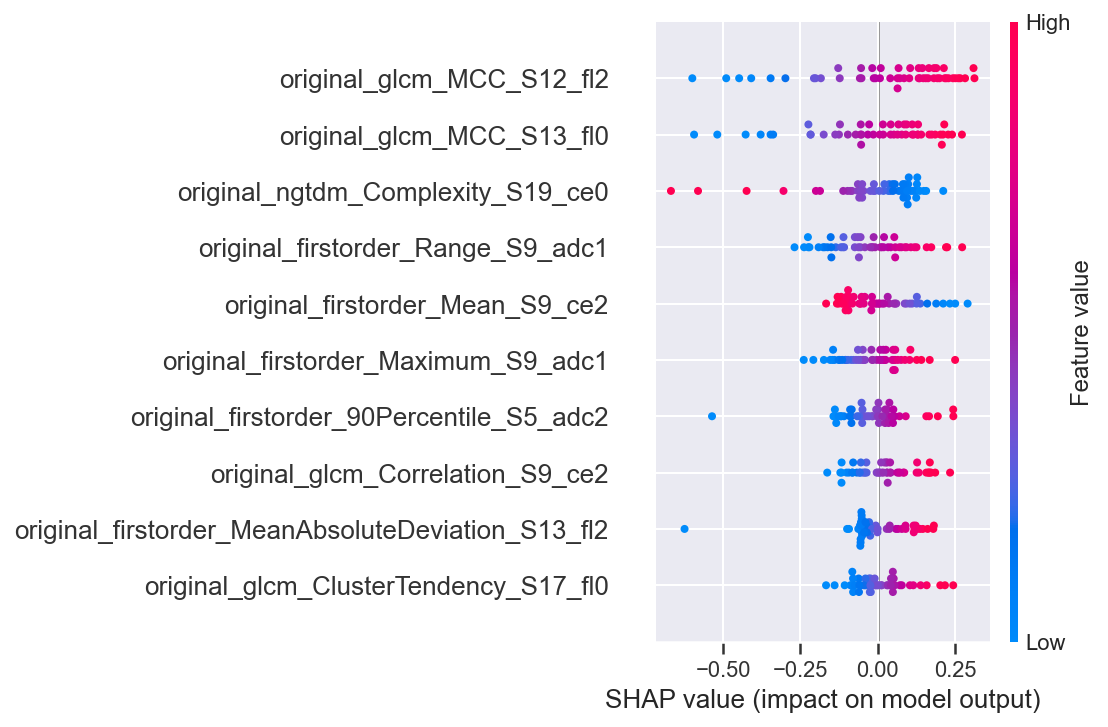


I. Top 10 radiomics for PTEN prediction

II. Top 10 radiomics for Survival prediction

III. Prediction AUC with different radiomics

IV. Percentage of different radiomics

*Figure S14: Significant feature analysis: (I) SHAP analysis with hierarchical clustering for PTEN prediction; (II) SHAP analysis with hierarchical clustering for Survival prediction; (III) Heatmap of prediction AUC across different radiomic feature categories; (IV) Distribution of radiomic feature types among all features (blue), the top 400 features ranked by PTEN prediction (orange), and the top 400 features ranked by survival prediction (green).*

*Table S3. Percentages of significant difference radiomics between different GBM regions*

| Modalities | Averaged percentage of patients having a significant difference for individual radiomics | | | Percentage of radiomics having a significant difference with at least one patient | | |
| --- | --- | --- | --- | --- | --- | --- |
|  | Necrotic vs. T1 | T1 vs. T2 | T2 vs. 2cm | Necrotic vs. T1 | T1 vs. T2 | T2 vs. 2cm |
| T1CE | 13.2% | 41.2% | 79.4% | 100% | 100% | 100% |
| FLAIR | 18.0% | 50.3% | 77.8% | 100% | 100% | 100% |
| ADC | 11.8% | 50.3% | 79.6% | 100% | 100% | 100% |

*Table S4. Mean and Standard Deviation of Fitting Parameters and Qualities for First Order Mean Intensity*

|  | Term | MGMT Positive (Mean $\pm$ Std) | MGMT Negative (Mean $\pm$ Std) |
| --- | --- | --- | --- |
| Parameters | K1 | -66.57 $\pm$ 39.59 | -69.23 $\pm$ 41.60 |
|  | K2 | 58.26 $\pm$ 43.65 | 53.81 $\pm$ 41.75 |
|  | L1 | 0.60 $\pm$ 0.19 | 0.57 $\pm$ 0.19 |
|  | L2 | -0.15 $\pm$ 0.42 | -0.22 $\pm$ 0.43 |
|  | X1 | 40.64 $\pm$ 17.51 | 37.22 $\pm$ 15.54 |
|  | X2 | 61.47 $\pm$ 20.22 | 58.35 $\pm$ 18.15 |
|  | b | 0.22 $\pm$ 0.22 | 0.27 $\pm$ 0.23 |
| Fitting Quality | R-Square | 0.68 $\pm$ 0.16 | 0.70 $\pm$ 0.16 |
|  | RMSE | 0.19 $\pm$ 0.05 | 0.18 $\pm$ 0.05 |

## I CLEAR Checklist

| Item | Reported (Y/N) | Section |
| --- | --- | --- |
| Imaging modality and sequence | Y | OVERVIEW OF RESEARCH APPROACH |
| Voxel size, slice thickness | Y | METHODS / Feature collection and selection |
| ROI segmentation method | Y | METHODS / Different tumor regions of GBM |
| Feature classes | Y | METHODS / Different tumor regions of GBM |
| Feature selection method | Y | METHODS / Feature collection and selection |
| Cross-validation strategy | Y | METHODS / Prediction Model |
| External validation cohort | Y | OVERVIEW OF RESEARCH APPROACH |
